# *PtrVINV2* is dispensable for cellulose synthesis but essential for salt tolerance in *Populus trichocarpa* Torr. and Gray

**DOI:** 10.1111/pbi.70022

**Published:** 2025-02-24

**Authors:** Shuang Zhang, Lina Cao, Danyang Chen, Ruhui Chang, Jiayu Cao, Qiaoyi Zhang, Yeling Qin, Guanjun Liu, Zhiru Xu

**Affiliations:** ^1^ State Key Laboratory of Tree Genetics and Breeding Northeast Forestry University Harbin China; ^2^ College of Life Science Northeast Forestry University Harbin China; ^3^ School of Forestry Northeast Forestry University Harbin China

**Keywords:** *Populus trichocarpa* Torr. and Gray, *PtrVINV2*, cellulose, hemicellulose, lignin, salt tolerance

## Abstract

Invertase (EC.3.2.1.26), a key enzyme in sucrose breakdown, is crucial for cellulose synthesis. However, the function of the vacuolar invertase (VINV) in woody plants remains unclear. In this study, transgenic lines of *Populus trichocarpa* Torr. and Gray were generated to investigate the role of *PtrVINV2* in wood formation and under high salinity stress. Compared to wild‐type (WT), VINV activity in the developing xylem of knockout lines was reduced, resulting in a decrease in lignin content and an increase in hemicellulose content, while cellulose content remained unaffected. These changes in structural carbohydrate content were accompanied by reductions in xylem width and fibre cell wall thickness. The overexpression lines of the developing xylem exhibited opposite trends. Transcriptome analyses of developing xylem indicated that the expression level of *PtrVINV2* affects the expression of genes involved in hemicellulose and lignin biosynthesis pathways, such as *AXS*, *UAMs*, *HCT*, *COMT*, *CAD* and *peroxidases*, while *CesA* expression remained unaffected. WGCNA analysis revealed that *Potri.001G219100*, *Potri.009G106600* and *Potri.002G081000* serve as ‘hub’ transcription factor genes within the structural/non‐structural carbohydrate modules of *PtrVINV2* transgenic lines, potentially involved in plant salt tolerance. Additionally, under 200 mmol/L NaCl treatment, the knockout lines exhibited increased salt sensitivity compared to WT. This increased sensitivity was accompanied by elevated activities of SOD, CAT and MDA, as well as higher sucrose content and reduced contents of glucose and fructose. The findings indicate that although *PtrVINV2* is not essential for cellulose synthesis, it enhances salt tolerance in poplar and presents a promising candidate gene for breeding salt‐tolerant poplar.

## Introduction

Invertases (INVs, EC.3.2.1.26) are also known as sucrases or β‐D‐fructofuranosidases. They catalyse the irreversible hydrolysis of sucrose into glucose and fructose, thereby playing a crucial role in carbon use (Rende *et al*., 2017). In plants, INVs constitute a multi‐gene family whose members are grouped into two subfamilies, acid invertases and neutral/alkaline invertases (NINVs), based on their optimal pH. Acidic invertases can be further classified into cell wall invertases (CWINVs) and vacuolar invertases (VINVs), depending on their subcellular localization (Haouazine‐Takvorian *et al*., 1997; Roitsch and Gonzalez, 2004; Sherson *et al*., 2003). Members of the NINV subfamily are typically localized in the cytoplasm, mitochondria and chloroplasts (Ji *et al*., 2005). VINV catalyses the hydrolysis of sucrose into two hexose molecules, thereby playing a crucial role in tissues with elevated hexose content. For example, the suppression of *SlVINV1* (*TIV1*) expression in tomato (*Solanum lycopersicum* L.) fruits, which accumulate both hexose and sucrose, results in the accumulation of sucrose instead of hexose (Klann *et al*., 1996). Additionally, sucrose is converted into glucose and fructose by VINV (doubled osmotic effect), which is associated with high VINV activity in many rapidly growing tissues. However, this effect is observed only when cells accumulate high concentrations of soluble sugars (SS) (Ruan *et al*., 2010). For example, in the elongating roots of *Arabidopsis thaliana* L., *AtVINV2* has been shown to regulate cell expansion through a non‐osmotic‐dependent pathway (Sergeeva *et al*., 2006). This is because sucrose and hexose contribute <2% to total osmotic pressure, so any change in VINV activity may have no effect on the osmotic effect (Lu *et al*., 2010).

Furthermore, in plants, *VINV* genes are involved in responding to both biotic and abiotic stresses. For instance, in grapes (*Vitis vinifera* L.), the promoter of the *EhvINVNMP* gene that encodes VINV is regulated by gibberellin but remains unresponsive to copper stress, drought, IAA, 6‐BA, ABA, ACC, MeJA and SA (Xu *et al*., 2023b). All six *VINV* genes in *Arachis hypogaea* L. are responsive to high salt stress (Mao *et al*., 2024). In *Cucumis sativus* L., overexpression of the *CsVI2* gene (encoding VINV) results in increased VINV activity and improved drought tolerance in seedlings (Chen *et al*., 2021). In *A. thaliana*, overexpression of the *CsINV5* gene (encoding VINV) of *Camellia sinensis* L. results in increased glucose and fructose levels and an altered osmotic potential, thus enhancing the cold tolerance of the transgenic plants (Qian *et al*., 2018). However, studies on the involvement of vacuolar invertase in biotic and abiotic stress responses in poplar remain limited, particularly under high salt stress. Extensive soil salinization severely affects the survival and growth of poplars, presenting a major challenge to wood production (Zhao *et al*., 2019). Osmotic stress is frequently encountered by plants in high‐salinity environments, where reduced soil water potential results in increased osmotic potential within root cells. This condition impairs water absorption capabilities and may lead to physiological leakage within the plant, culminating in physiological drought (Cheng *et al*., 2019). With the rapid advancement of genetic engineering technology, substantial progress has been achieved in developing transgenic *Populus* varieties that exhibit drought and salt‐alkali resistance. For example, transgenic *Populus alba* L. × *P. berolinensis* K. Koch plants that overexpress the *JERF36s* gene and were grown for four years exhibited superior height and diameter growth rates compared to WT, along with significantly enhanced salt tolerance (Ding *et al*., 2020). The cultivation of transgenic *Populus* varieties with improved salt tolerance offers an effective approach to enhancing the survival and productivity of these trees in saline‐alkaline environments. Therefore, it is necessary to investigate the function of vacuolar invertase in poplar under high‐salt conditions.

Although VINV is a subfamily of the INV family and functions as a key enzyme in sucrose hydrolysis in plants, its activity has rarely been linked to the synthesis of structural carbohydrates (SCs) such as cellulose. Most studies suggest that NINVs and CWINVs are more likely to provide carbon sources for cellulose synthesis. For example, in *Populus tremula* L. × *tremuloides* Michx., inhibition of the cytoplasmic invertase *CINV12* resulted in a 38%–55% decrease in NINV activity and a 9%–13% reduction in crystalline cellulose content in wood tissue (Rende *et al*., 2017). In *P. trichocarpa*, knockout of the *PtrNINV12* gene resulted in significant reductions in cellulose and hemicellulose contents, accompanied by decreased fibre cell wall thickness (Zhang *et al*., 2023). Seedlings of *Arabidopsis* cytoplasmic invertase mutant *cinv1cinv2* displayed a loss of anisotropic growth characteristics. These phenotypic changes were associated with reduced cellulose content, abnormal cellulose synthase complexes, and alterations in cell wall matrix polysaccharides (Barnes and Anderson, 2018). In *Arabidopsis*, heterologous expression of *PhCWINV1*, *PhCWINV4* or *PhCWINV7* from *Phyllostachys edulis* (Carrière) J. Houz. increased the biomass of transgenic plants, indicating that *PhCWINVs* can promote internode elongation (Guo *et al*., 2020). In *Triticum aestivum* L., overexpression of the *CWINV* gene *TaCWI‐B1* resulted in a significant increase in cell wall thickness, pectin content and cellulose content. Moreover, TaCWI‐B1 was found to directly interact with α‐galactosidase (TaGAL) to confer resistance to pests and diseases (Lv *et al*., 2023). However, few studies in other plants have shown that VINVs are involved in the synthesis of SCs, such as cellulose. For example, in *Gossypium hirsutum* L., overexpression of *GhVINV1* significantly increased VINV activity, accompanied by a substantial increase in cellulose content. Conversely, inhibition of *GhVINV1* expression resulted in a significant decrease in cellulose content (Lu *et al*., 2010; Wang *et al*., 2014). In *Arabidopsis*, overexpression of the *Populus tomentosa* Carrière vacuolar invertase *PtoVINV3* gene increased trichome density, indicating that PtoVINV3 plays a key role in the development of poplar catkin fibres (Yang *et al*., 2023).

Forests act as extensive and persistent carbon sinks, capturing about one‐quarter of the carbon dioxide released from human activities. This carbon is stored in wood and, combined with soil‐bound carbon, forms a long‐term terrestrial carbon sink (Lal, 2008; Pan *et al*., 2011). Wood is recognized as one of the most sustainable raw materials, and considerable attention has been directed toward the development of novel wood‐derived materials and renewable fuels. *Populus*, as a model woody species, has been extensively studied for the modification of gene expression to alter wood properties, with the goal of increasing cellulose content and reducing the resistance of cell wall components to saccharification (such as lignin) (Bryant *et al*., 2020). Consequently, the enhancement of wood properties in *Populus* through genetic modification has emerged as a research focus for forestry scientists (Chudy *et al*., 2019; Shooshtarian *et al*., 2018). *P. trichocarpa*, a fast‐growing and highly adaptable poplar species, produces wood through typical secondary growth. It is one of the most promising sources of woody biomass among various tree species. Its clear genetic background and ease of genetic transformation have made it a model plant for genetic engineering. Therefore, *P. trichocarpa* is an ideal material for studying lignocellulose and its formation (Tuskan *et al*., 2006). The carbon used in wood formation is derived from sucrose produced during photosynthesis (Turgeon, 1996). The transformation of sucrose into wood involves a complex metabolic pathway, wherein the disaccharide sucrose must be hydrolyzed into monosaccharides for further utilization. This hydrolysis is facilitated by the enzymatic activity of INVs (Samac *et al*., 2015). In various plants, NINVs and CWINVs have been demonstrated to provide carbon for cellulose biosynthesis (Barnes and Anderson, 2018; Lv *et al*., 2023; Rende *et al*., 2017; Zhang *et al*., 2023). However, few studies have investigated the involvement of VINVs in the synthesis of SCs, such as cellulose, particularly in woody plants.

Hence, this study investigated the role of *PtrVINV2* in poplar wood formation and under high salinity stress using transgenic technology, wood anatomy, physiological and biochemical assays and transcriptome sequencing. It assesses whether VINV's hydrolytic activity contributes to the carbon supply for lignocellulose synthesis and provides new insights for developing germplasm resources with high lignocellulose content. In addition, VINVs, as essential enzymes for sucrose hydrolysis in vacuolar tissues, are crucial for regulating intracellular osmotic pressure. Therefore, determining whether the sucrose hydrolytic activity of VINVs contributes to salt tolerance in *P. trichocarpa* will yield promising candidate genes for the development of salt‐tolerant varieties.

## Results

### PtrVINV2 localizes in the vacuolar membrane and its transcript levels are significantly correlated with cellulose, hemicellulose and lignin contents

**Figure 1:**
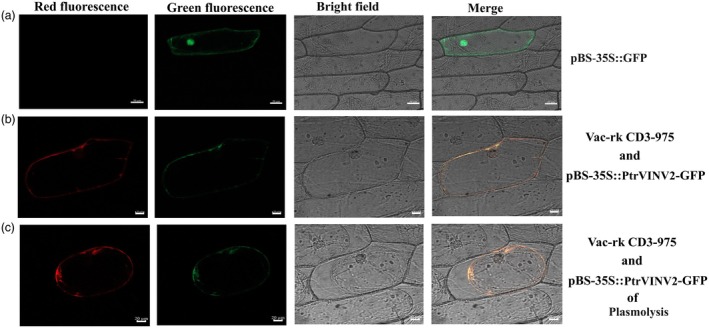
Subcellular localization of PtrVINV2 protein. Red fluorescence, green fluorescence, bright field and merged images of (a) pBS‐35S::GFP, bars = 50 μm, (b) Vac‐rk CD‐975 (vacuolar membrane localization vector) and pBS‐35S::PtrVINV2‐GFP, bars = 20 μm, (c) Vac‐rk CD‐975 and pBS‐35S::PtrVINV2‐GFP of plasmolysis, bars = 20 μm.

A previous study (Zhang *et al*., 2023) found that the transcript level of *PtrVINV2* was positively correlated with cellulose content, and negatively correlated with hemicellulose, lignin and starch contents (Figure S1). Because cellulose, hemicellulose and lignin are major products of secondary xylem growth, those results may indicate that *PtrVINV2* is involved in the development of the secondary xylem. Studies on other plants have shown that VINVs are typically localized in the vacuolar membrane (Haouazine‐Takvorian *et al*., 1997; Roitsch and Gonzalez, 2004; Sherson *et al*., 2003). To experimentally determine the subcellular localization of PtrVINV2, the pBS‐35S::PtrVINV2‐GFP, Vac‐rk CD3‐975 (positive control) and pBS‐35S::GFP (negative control) vectors were transiently expressed in onion epidermal cells. As shown in Figure 1, the green fluorescence signal of pBS‐35S::PtrVINV2‐GFP overlapped with the red fluorescence of Vac‐rk CD3‐975. After plasmolysis, the fluorescence signal shifted in accordance with the movement of the central vacuole. This showed that PtrVINV2 localizes to the vacuolar membranes, confirming its classification as a VINV.

### Overexpression or knockout of *PtrVINV2* affected VINV activity

**Figure 2:**
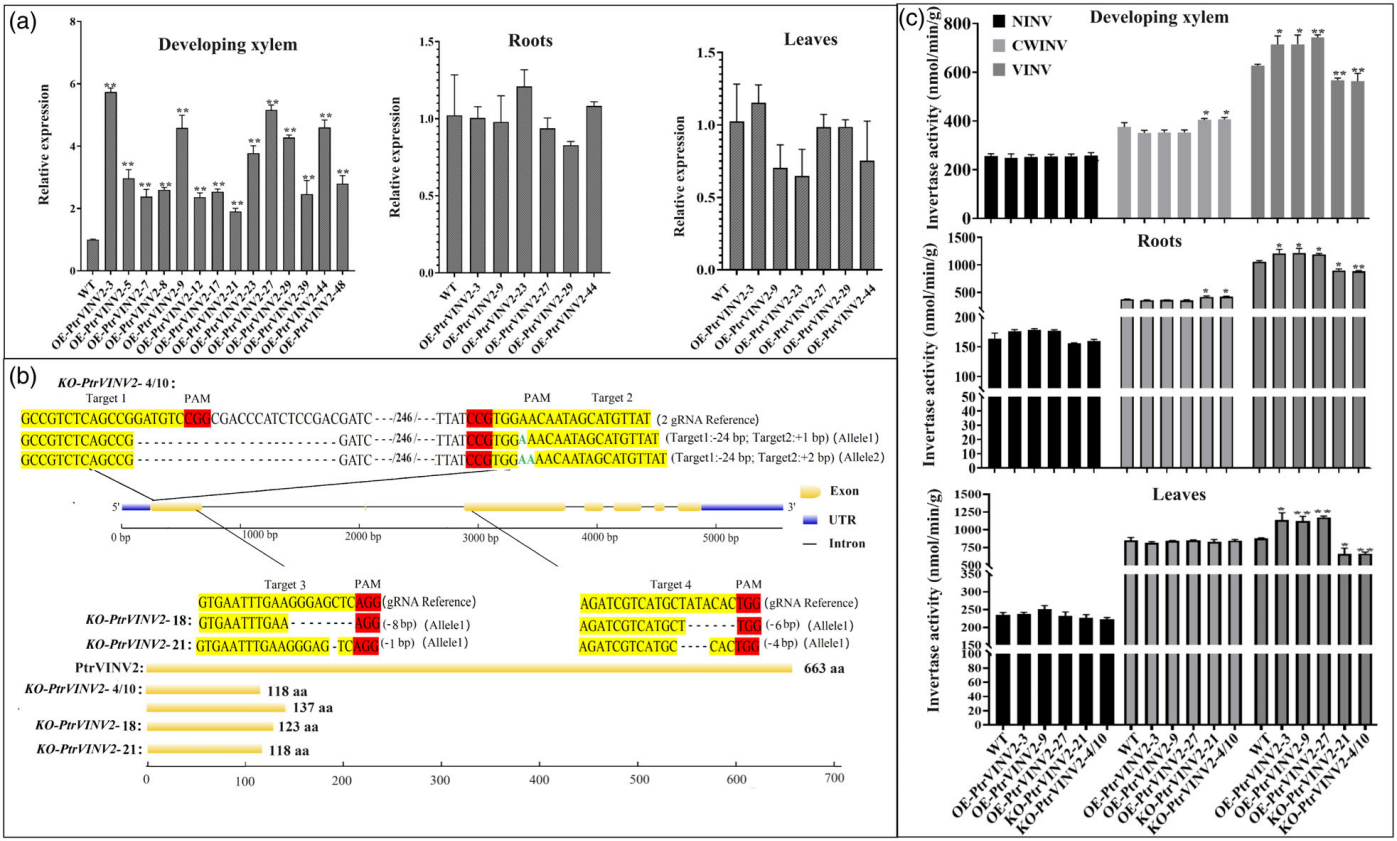
Identification of *PtrVINV2*‐overexpression and ‐knockout lines. (a) Relative transcript levels of *PtrVINV2* in developing xylem, roots and leaves of *PtrVINV2*‐overexpression lines (OE‐*PtrVINV2*). (b) Editing types of *PtrVINV2* gene knockout lines (KO‐*PtrVINV2*), with target sites highlighted in yellow, PAM sites highlighted in red, insertions shown in green font, ‘‐’ indicating deletions, ‘‐‐/bp/‐‐’ indicating omitted nucleotides and editing methods and allele types annotated in parentheses. (c) Enzymatic activities in developing xylem, roots and leaves in OE‐*PtrVINV2* and KO‐*PtrVINV2* lines. Asterisks denote significant differences (*t*‐test, *P < 0.05, **P < 0.01).

To investigate the function of VINV during wood formation, we generated OE‐*PtrVINV2* lines under the control of the DX15 promoter (developing xylem promoter), and KO‐*PtrVINV2* using CRISPR/Cas9 technology. The results of qRT‐PCR showed a significant upregulation of the *PtrVINV2* gene in the developing xylem of OE‐*PtrVINV2* lines. Transcriptional levels were elevated by 2‐fold to 5.8‐fold, with the most significant increases detected in OE‐*PtrVINV2*‐3 (5.8‐fold), OE‐*PtrVINV2*‐9 (4.6‐fold) and OE‐*PtrVINV2*‐27 (5.2‐fold). Subsequently, the transcript levels of *PtrVINV2* were assessed in the leaves and roots of the top six lines with the highest fold changes in the transcript level of *PtrVINV2* in the developing xylem. The results revealed expression variations ranging from 0.64‐fold to 1.14‐fold (Figure 2a). These results indicated that overexpression of *PtrVINV2* resulted in increases in its transcript levels almost exclusively in the developing xylem, with minimal effects on its transcript levels in leaves and roots.

The editing types of the knockout lines are illustrated in Figure 2b. KO‐*PtrVINV2*‐4/10 displayed two editing types (Allele1 and Allele2), constituting biallelic mutations, while KO‐*PtrVINV2*‐18 and KO‐*PtrVINV2*‐21 displayed a single editing type (Allele1), indicative of a homozygous mutation. In the KO‐*PtrVINV2*‐4/10 line, Allele1 and Allele2 had a deletion of 24 nucleotides at the Target1 sequence, while Allele1 had one inserted ‘A’ nucleotide at the Target2 sequence and Allele2 had one inserted ‘AA’ nucleotide at the Target2 sequence. These deletions and insertions led to premature translation termination of Allele1 and Allele2 after the 118nd and 137nd amino acids, respectively. The editing type of KO‐*PtrVINV2*‐18 involved a deletion of one ‘GGGAGCTC’ nucleotide at the Target3 sequence and a deletion of six nucleotides (ATACAC) at the Target4 sequence, resulting in translation termination after the 123nd amino acid. In the KO‐*PtrVINV2*‐21 line, a single ‘C’ nucleotide was deleted at the Target3 sequence, along with a deletion of four nucleotides (TATA) at the Target4 sequence, leading to translation termination after the 118nd amino acid. These results indicated that the KO‐*PtrVINV2*‐4/10, KO‐*PtrVINV2*‐18 and KO‐*PtrVINV2*‐21 lines were successful gene knockouts suitable for further study.

To further assess the effects of *PtrVINV2*‐overexpression and ‐knockout on INV activity, the activities of three types of invertases (NINV, CWINV and VINV) were measured. Unexpectedly, dwarfism and chlorosis were observed in the KO‐*PtrVINV2*‐18 line compared to the WT, while no such phenotypes were detected in the KO‐*PtrVINV2*‐21 and KO‐*PtrVINV2*‐4/10 lines (Figure S2). This phenomenon is hypothesized to result from the disruption of other gene expression caused by the T‐DNA insertion site. Consequently, INV activity in the KO‐*PtrVINV2*‐18 line was not measured, and no further analysis will be performed in future experiments. As shown in Figure 2c, compared to the WT, VINV activity in the developing xylem, roots and leaves of the OE‐*PtrVINV2* lines was significantly increased, while a significant reduction was observed in the KO‐*PtrVINV2* lines. Additionally, NINV activity was unchanged in all tissue types in the OE‐*PtrVINV2* and KO‐*PtrVINV2* lines. However, CWINV activity was significantly increased in the developing xylem and roots of the KO‐*PtrVINV2* lines. In summary, overexpression of the *PtrVINV2* gene in developing xylem led to a significant increase in VINV activity. Conversely, the knockout lines demonstrated a significant decrease in VINV activity in the developing xylem.

### *PtrVINV2* promotes secondary xylem development in *P. Trichocarpa*

The OE‐*PtrVINV2* and KO‐*PtrVINV2* lines were cultivated for 3 months and their growth phenotypes were investigated. As shown in Figure S2, the height, leaf number, internode number, diameter and internode length of the OE‐*PtrVINV2* lines are comparable to those of the WT. The diameters and ground diameters of the 16th internode in the KO‐*PtrVINV2*‐21 and KO‐*PtrVINV2*‐4/10 lines were significantly greater than those of the WT, with no significant differences observed in other growth indices compared to WT. The results indicate that the knockout of the *PtrVINV2* gene promotes the development of stem internode diameter in *P. trichocarpa*, whereas overexpression of the *PtrVINV2* gene has a minimal effect on stem internode diameter growth.

**Figure 3:**
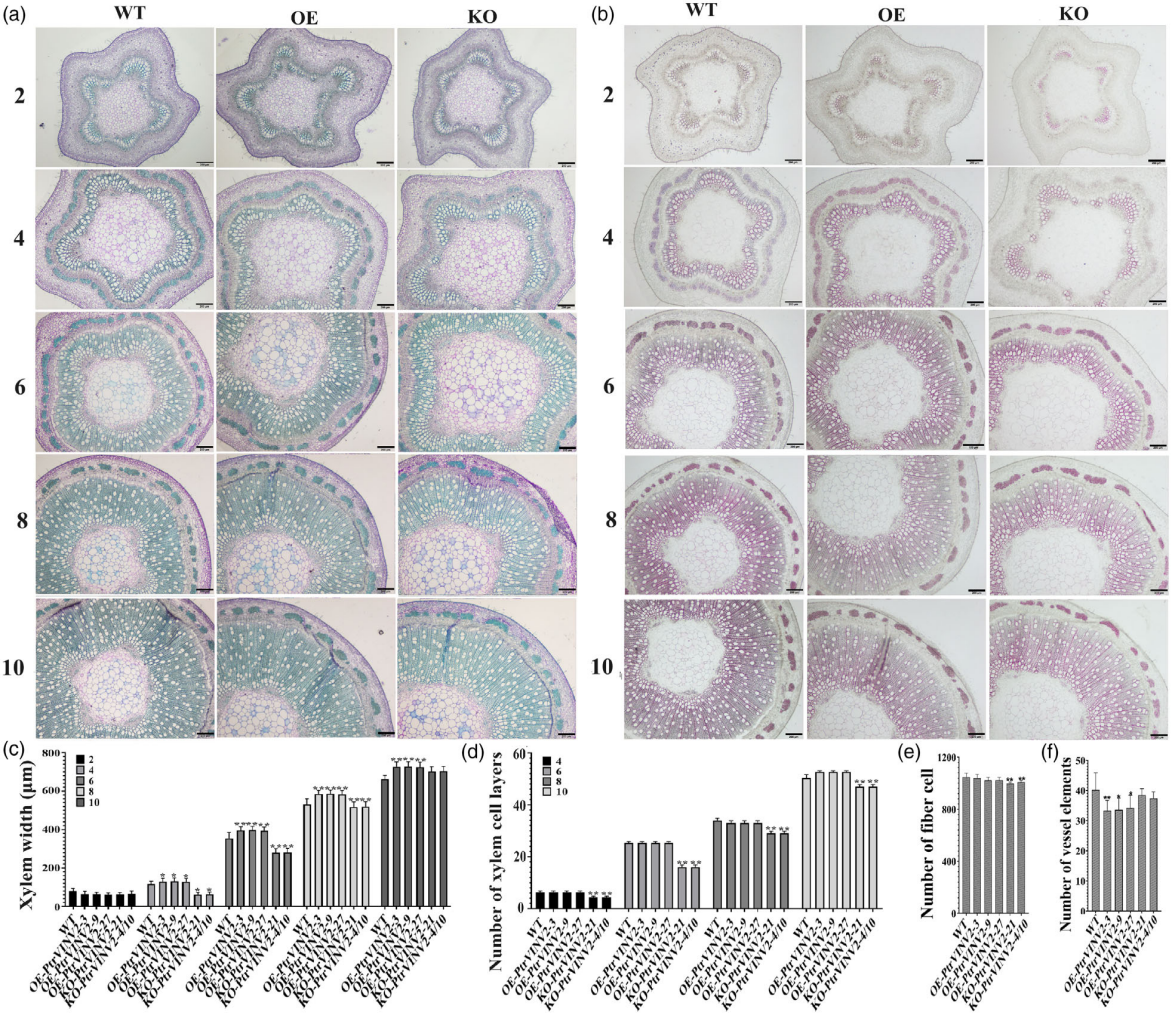
Analysis of histochemical staining in *PtrVINV2*‐overexpression and ‐knockout lines. (a) Toluidine‐blue staining. (b) Phloroglucinol‐HCl staining. (c) Width statistics of the xylem. (d) Cell layer statistics of the xylem. 2, 4, 6, 8 and 10 represent 2nd, 4th, 6th, 8th and 10th internodes, respectively. Bars = 200 μm. (e) The number of fibre cells within the same cross‐sectional area at the 10th internode. (f) The number of vessel elements within an equivalent cross‐sectional area at the 10th internode. Asterisks indicate significant differences (*P < 0.05, **P < 0.01) as determined by *t*‐test.

On the basis of the differences in stem diameter among transgenic lines, the tissue morphology and xylem structure of the 2nd (2 IN), 4th (4 IN), 6th (6 IN), 8th (8 IN) and 10th (10 IN) internodes of 3‐month‐old transgenic plants were further analysed by toluidine blue staining and phloroglucinol‐HCl staining. As shown in Figure 3, toluene blue staining revealed a significant increase in xylem width at 4 IN, 6 IN, 8 IN and 10 IN in the OE‐*PtrVINV2* lines compared to the WT, despite no change in the number of xylem cell layers. Conversely, both the xylem width and cell layer count at 4 IN, 6 IN and 8 IN in the KO‐*PtrVINV2* lines were significantly reduced compared to the WT. The pith diameter in the KO‐*PtrVINV2* lines was enlarged relative to the WT (Figure 3a,c,d). Furthermore, a comparison of transgenic and WT plants within cross‐sections of equal area indicated significantly fewer vessel elements in OE‐*PtrVINV2* lines and reduced fibre cells in KO‐*PtrVINV2* lines (Figure 3e,f; Figure S3). The phloroglucinol‐HCl staining intensity indicated the lignin content. In this study, from 4 IN onwards, the staining intensity was lower in KO‐*PtrVINV2* lines compared to WT, but was similar between WT and OE‐*PtrVINV2* lines (Figure 3b). These results suggested that *PtrVINV2*‐overexpression and ‐knockout primarily influenced secondary xylem development at 4 IN, 6 IN, 8 IN and 10 IN.

**Figure 4:**
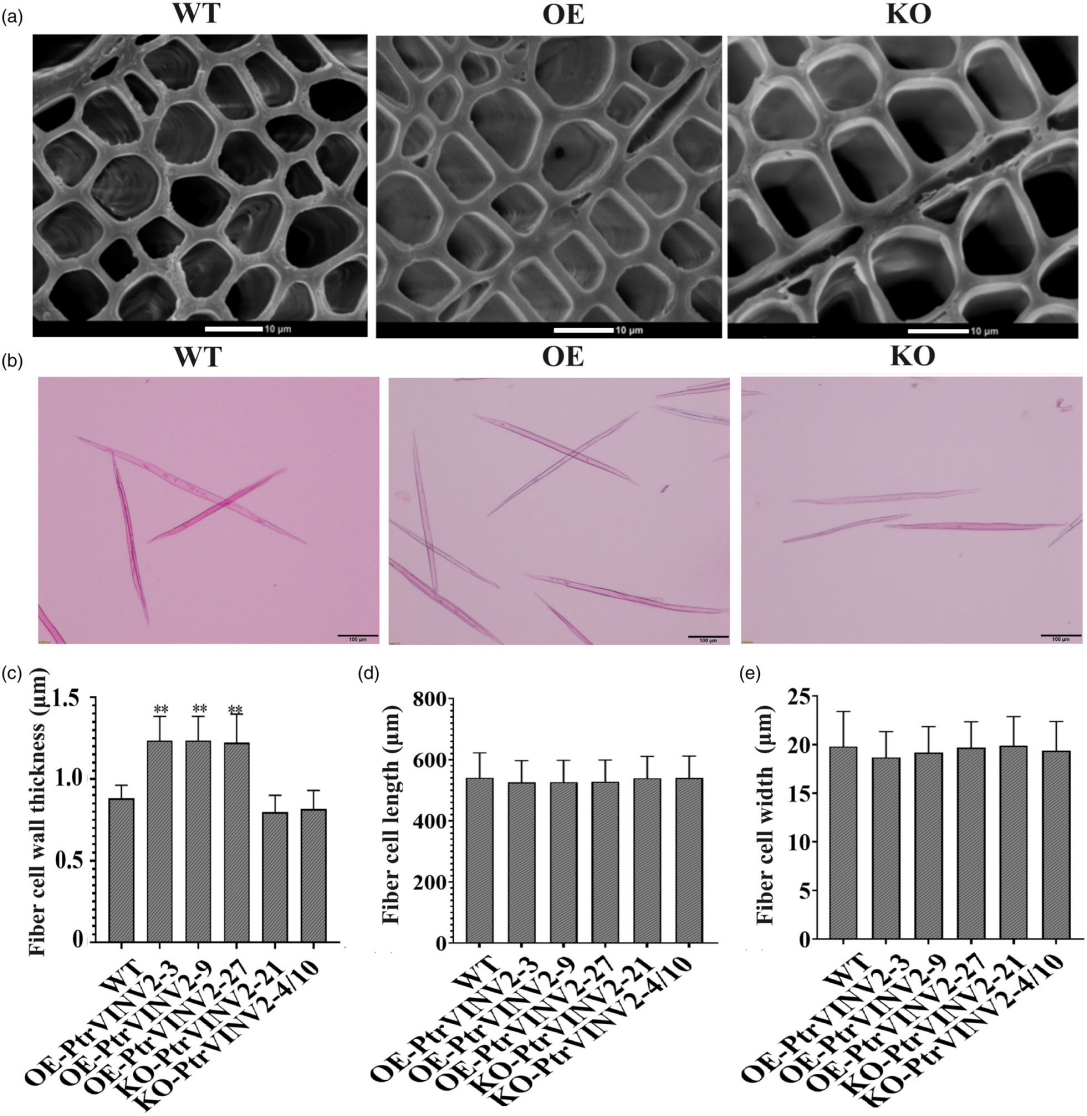
Micrographs of mature fibre cells in *PtrVINV2*‐overexpression and ‐knockout lines, and statistical comparison of fibre cell characters. (a) Cell wall thickness of fibre cells, bars = 10 μm. (b) Length and width of fibre cells, bars = 100 μm. Statistical analysis of (c) fibre cell wall thickness, (d) fibre cell length and (e) fibre cell width. Asterisks indicate significant differepnces (*P < 0.05, **P < 0.01) as determined by *t*‐test.

To assess whether changes in xylem width correlate with fibre cell wall thickness, fibre cell characteristics of 10 IN were analysed. Fibre cell wall thickness observations revealed a significant increase in the OE‐*PtrVINV2* lines compared to WT, while the KO‐*PtrVINV2* lines exhibited a nonsignificant thinning trend (Figure 4a,c). Neither the knockout nor overexpression of *PtrVINV2* impacted fibre cell length or width (Figure 4b,d,e). In summary, alterations in fibre cell wall thickness in *PtrVINV2* transgenic plants contribute to the observed changes in xylem width.

### *PtrVINV2* regulates lignin and hemicellulose contents without affecting cellulose content

**Figure 5:**
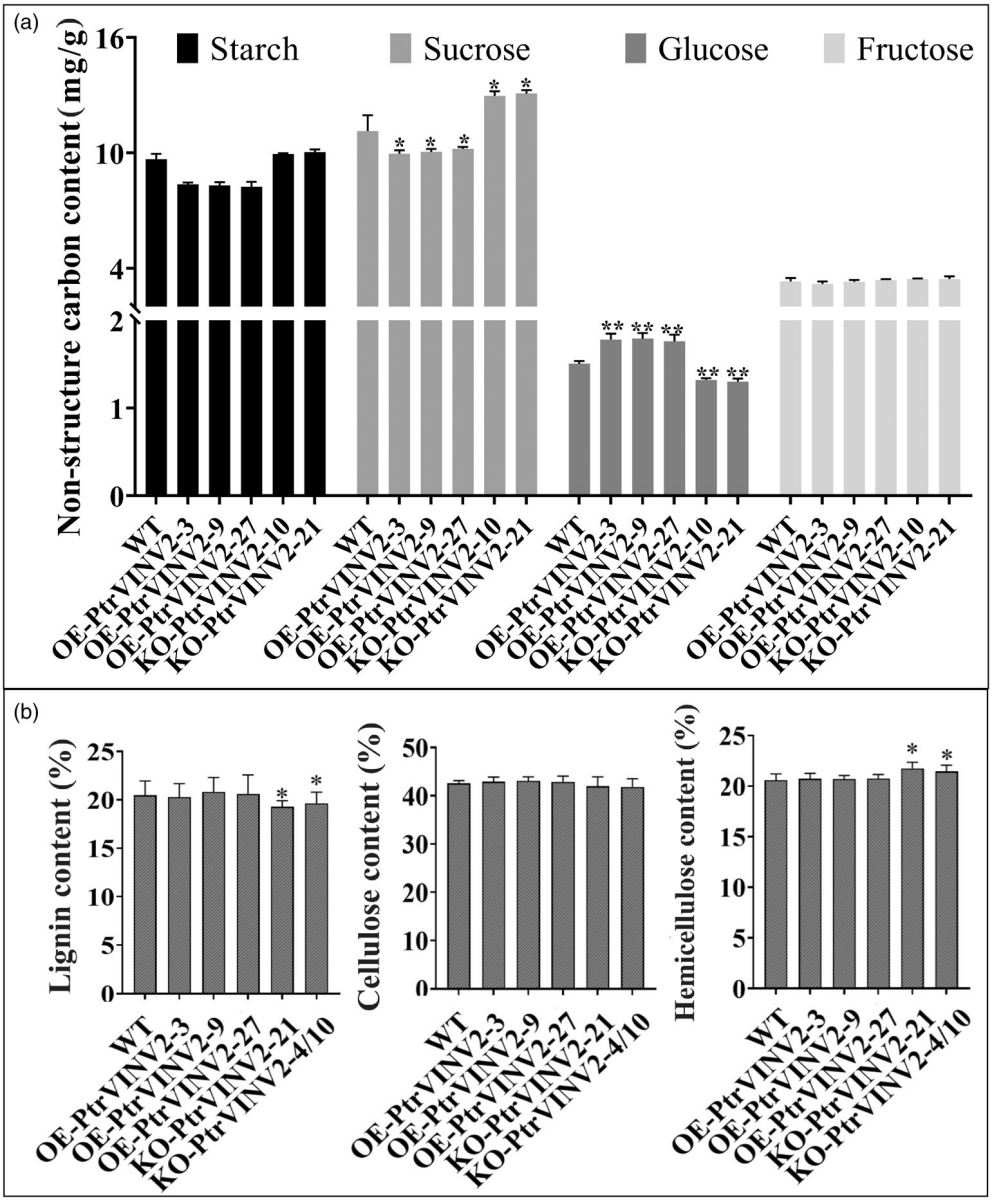
Carbohydrate contents in *PtrVINV2*‐overexpression and ‐knockout lines. (a) Non‐structural carbohydrate contents (starch, sucrose, glucose, fructose). (b) Structural carbohydrate contents (lignin, cellulose, hemicellulose). Asterisks indicate significant differences (*t*‐test, *P < 0.05, **P < 0.01).

The contents of SCs and non‐structural carbohydrates (NSCs) in stem samples were compared among the transgenic plants and the WT to investigate whether they were associated with changes in microstructure in the transgenic plants. As shown in Figure 5a, in NSC components, the OE‐*PtrVINV2* lines demonstrated a significant reduction in sucrose content and an increase in glucose content compared to the WT. In contrast, the KO‐*PtrVINV2* lines showed elevated sucrose content and reduced glucose content. In addition, no significant changes in SC components were detected in OE‐*PtrVINV2* lines compared to the WT. KO‐*PtrVINV2* lines showed a significant increase in hemicellulose content and a reduction in lignin content, with cellulose content remaining stable (Figure 5b). It was concluded that the changes in the contents of lignin and hemicellulose in KO‐*PtrVINV2* lines might be the main factors related to the changes in secondary xylem development.

### Changes in transcript levels of genes involved in carbon metabolism in the transgenic lines

To explore the molecular mechanisms underlying the changes in secondary structure, the developing xylem (the active region of the secondary xylem) was isolated for transcriptome sequencing. The results of principal component analyses and hierarchical clustering analyses indicated excellent repeatability within groups and independence among groups during clustering (Figure S4A,B). Correlation analyses also revealed high intra‐group correlations and lower inter‐group correlations (Figure S4C). These results collectively demonstrated the high quality of the transcriptome dataset, which facilitated subsequent analyses. To determine the effects of *PtrVINV2*‐overexpression (OEV) and *PtrVINV2*‐knockout (KOV) on gene transcript levels in the developing xylem, DESeq2 software was used to identify DEGs (with the WT as the control group). In the comparison between the OEV and WT groups (OEV vs. WT), 1499 DEGs were significantly upregulated and 652 DEGs were significantly downregulated. Similarly, in the comparison between the KOV and WT groups (KOV vs. WT), 1231 DEGs were significantly upregulated and 614 DEGs were significantly downregulated (Figure S5A). To functionally categorize the identified DEGs, KEGG enrichment analyses were conducted separately for the DEGs from the OEV and KOV groups. The KEGG enrichment results (Figure S5B) showed that DEGs from both the OEV vs. WT and KOV vs. WT comparisons were significantly enriched in pathways such as starch and sucrose metabolism, plant hormone signal transduction and phenylpropanoid biosynthesis. The DEGs from the OEV vs. WT comparison were significantly enriched in the amino sugar and nucleotide sugar metabolism pathway. The results of the KEGG enrichment analyses indicated that the *PtrVINV2*‐overexpression and ‐knockout significantly influenced the transcription levels of carbon metabolism‐related pathways.

**Figure 6:**
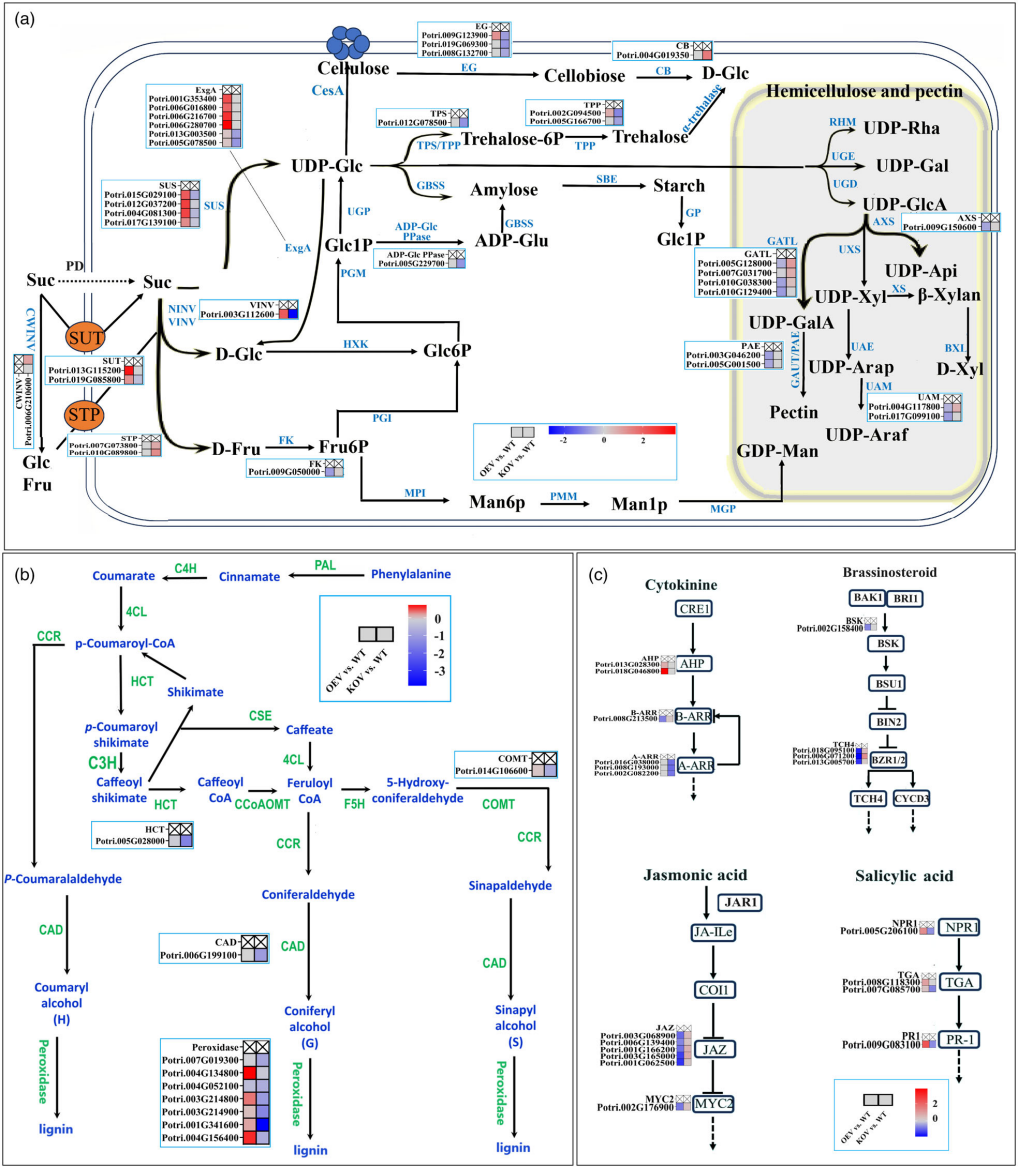
Expression patterns of genes in *PtrVINV2* transgenic plants. (a) Expression patterns of genes related to sucrose unloading, transport, metabolism and synthesis pathways of structural and non‐structural carbohydrates in *PtrVINV2* transgenic plants. The heatmap was constructed using Log_2_FC values of DEGs. Suc (sucrose), D‐Glc (D‐glucose), D‐Fru (D‐fructose), Fru6P (fructose 6‐phosphate), Man6P (mannose 6‐phosphate), Man1P (mannose 1‐phosphate), GDP‐Man (GDP‐Mannose), Glc6P (glucose 6‐phosphate), Glc1P (glucose 1‐phosphate), UDP‐Glc (UDP‐Glucose), Trehalose‐6p (trehalose 6‐phosphate), UDP‐Rha (UDP‐rhamnose), UDP‐Gal (UDP‐galactose), UDP‐GlcA (UDP‐glucuronate), UDP‐Api (UDP‐apiose), UDP‐Xyl (UDP‐xylose), UDP‐GalA (UDP‐galacturonate), UDP‐Arap (UDP‐pyranose‐arabinose), UDP‐Araf (UDP‐furanose‐arabinose), D‐Xyl (D‐xylose), PD (plasmodesmus), CWINV (cell wall invertase), NINV (neutral/alkaline invertase), VINV (vacuole invertase), SUT (sucrose‐H^+^symporters), STP (sugar transport proteins), SUS (sucrose synthase), CesA (cellulose synthase), ExgA (glucan endo‐1,3‐β‐glucosidase), HXK (hexokinase), FK (fructokinase), PGI (phosphoglucose isomerase), PGM (glucose phosphoglucomutase), UGP (UDP‐glucose pyrophosphorylase), MPI (mannose 6‐phosphate isomerase), PMM (phosphomannomutase), MGP (mannose‐1‐phosphate guanylyltransferase), ADP‐Glc PPase (glucose‐1‐phosphate adenylyltransferase), GBSS (granule bound starch synthase), TPS (trehalose 6‐phosphate synthase), TPP (trehalose 6‐phosphate phosphatase), EG (endoglucanase), CB (β‐glucosidase), SEB (branching enzyme), GP (glycogen phosphorylase), TREH (α‐trehalase), RHM (multifunctional rhamnose biosynthesis enzyme), UGE (UDP‐glucose 4‐epimerase), UGD (UDP‐Glc dehydrogenase), AXS/UXS (UDP‐glucuronate decarboxylases), GATL (galacturonosyl transferase‐like), XS (xylan synthase), UAE (UDP‐Ara 4‐epimerase), BXL (β‐D‐xylosidase), UAM (UDP‐arabopyranose mutase), GAUT (α‐1, 4‐galacturonosyltransferase), PAE (pectin acetylesterase). (b) Differential expression of genes related to lignin synthesis in *PtrVINV2* transgenic plants. The heatmap was constructed using Log_2_FC values of DEGs. PAL (phenylalanine ammonialyase), C4H (cinnamate 4‐Hydroxylase), 4CL (4‐coumarate‐CoA ligase), CCR (cinnamoyl‐CoA reductase), HCT (shikimate O‐hydroxy cinnamoyl transferase), C3H (coumarate 3‐hydroxylase), CSE (caffeoyl shikimate esterase), CCoAOMT (caffeoy‐l CoA3‐O‐methyltransferase), F5H (ferulate5‐hydroxylase), COMT (caffeic acid‐3‐O‐methyltransferase), CAD (cinnamoyl alcohol dehydrogenase). (c) Differential expression of genes involved in hormone signalling pathways in *PtrVINV2* transgenic plants. The heatmap was generated using Log_2_FC values of DEGs. AUX1 (auxin influx carrier 1), TIR1 (transport inhibitor response 1), IAA (auxin/indole‐3‐acetic acid), ARF (auxin response factor), GH3 (gretchen hagen 3), SAUR (small auxin up RNA), CRE1 (cytokinin receptor 1), AHP (histidine phosphotransfer protein), B‐ARR (type‐B response regulator), A‐ARR (type‐A response regulator), PYL (abscisic acid receptor protein), PP2C (protein phosphatase 2C), SnRK2 (positive regulator SNF1‐associated protein kinase 2), ABF (ABRE binding factor), NPR1 (non‐expressor of pathogenesis‐related genes 1), TGA (transcription factor TGA), PR1 (pathogenesis‐related protein 1). The scale bar is shown in the figure.

To investigate the mechanisms underlying changes in hemicellulose and lignin contents in *PtrVINV2* transgenic plants, expression pattern analysis was conducted for the DEGs enriched in starch and sucrose metabolism pathways, amino sugar and nucleotide sugar metabolism and those involved in phenylpropanoid biosynthesis pathways. As shown in Figure 6a, the expression levels of the *PtrVINV2* gene, whether increased or decreased, have minimal impact on the genes associated with cellulose synthesis and degradation pathways. For instance, the expression levels of *CesAs* remained unchanged in both OEV and KOV, while *EGs* were upregulated in OEV and *CB* was upregulated in KOV. Furthermore, in the hemicellulose and pectin synthesis pathways, *AXS*, *GATLs*, *PAEs* and *UAMs* were downregulated in OEV. In the trehalose synthesis pathway, *TPS* and *TPPs* were downregulated in KOV. In the apoplast pathway, *SUTs* were upregulated in OEV, whereas *STPs* were upregulated in KOV. Changes in *PtrVINV2* expression also alter the expression patterns of genes involved in sucrose degradation. For instance, *CWINV* was upregulated in KOV, while *SUSs* were upregulated in OEV. Additionally, as shown in Figure 6b, in the lignin biosynthesis pathway, the expression of *HCT*, *COMT* and *CAD* genes was downregulated in KOV, meanwhile most *peroxidase* genes were upregulated in OEV and downregulated in KOV. In conclusion, the *PtrVINV2*‐overexpression and ‐knockout significantly influenced the expression patterns of genes related to hemicellulose and lignin synthesis pathways, leading to changes in their respective contents.

Based on the KEGG enrichment results, an expression pattern analysis was conducted for the DEGs enriched in plant hormone signal transduction pathways. In the cytokinin signal transduction pathway, *AHPs* were upregulated, whereas *B‐ARR* was downregulated in OEV. Simultaneously, *A‐ARRs* were downregulated in KOV. In the brassinosteroid signal transduction pathway, *BSK* and *TCH4* genes were downregulated in OEV while upregulated in KOV. In the jasmonic acid signal transduction pathway, *JAZs* and *MYC2* genes were downregulated in OEV while upregulated in KOV. In the salicylic acid signal transduction pathway, *NPR1*, *TGAs* and *PR‐1* genes were upregulated in OEV while downregulated in KOV (Figure 6c). The above results indicated that the *PtrVINV2*‐overexpression and ‐knockout influenced cytokinin, brassinosteroid, jasmonic acid and salicylic acid signalling pathways.

### ‘Hub’ genes in structural and non‐structural carbohydrate modules of *PtrVINV2* transgenic plants

**Figure 7:**
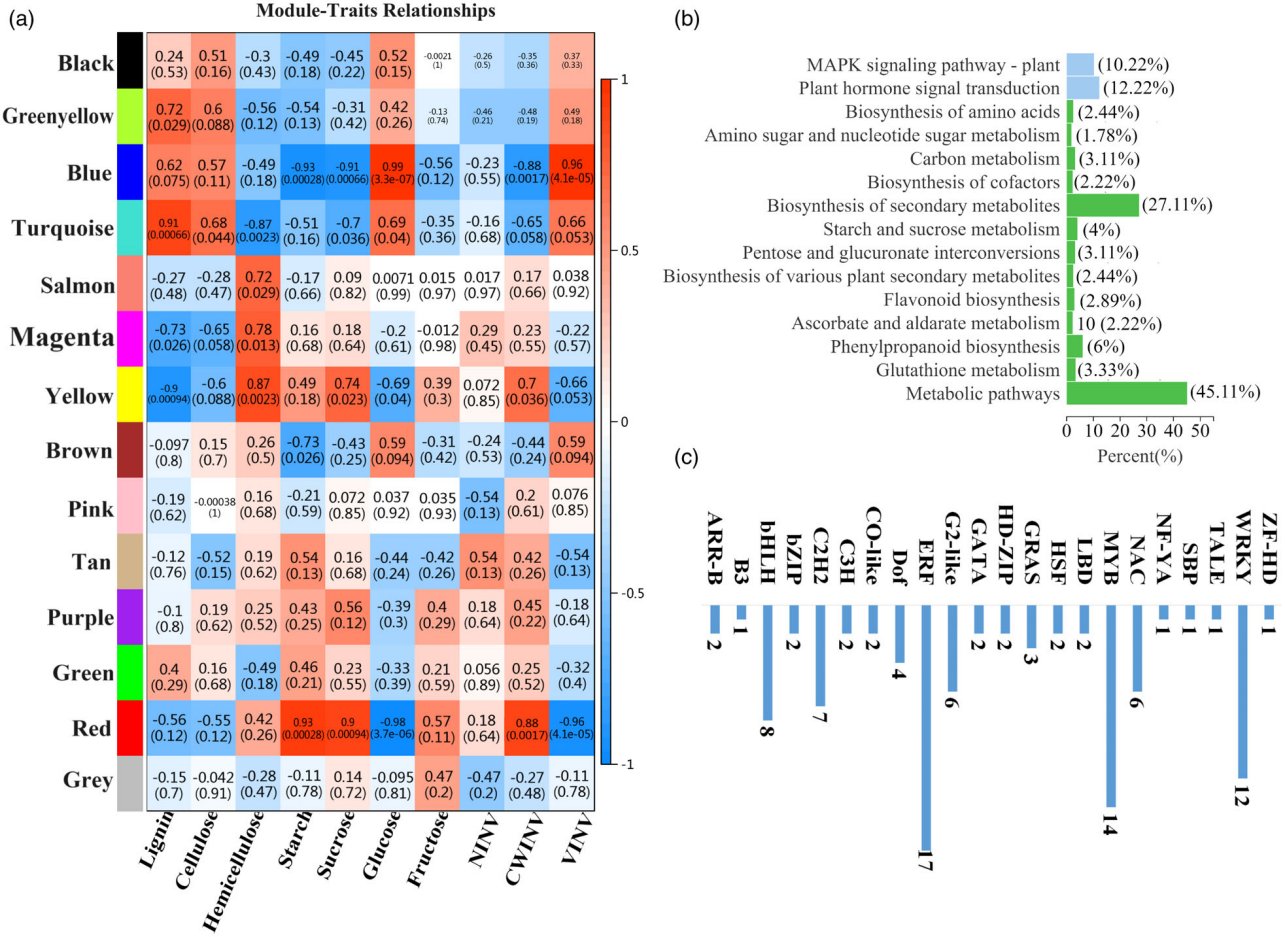
Joint analysis of transcriptome data and physiological indices in *PtrVINV2* transgenic plants. (a) WGCNA analysis of DEGs and physiological indices. X axis represents physiological phenotypes; Y axis represents different gene module hierarchical clusters. Numbers within modules indicate the correlation (significance) between modules and various physiological indices, with blue indicating a stronger negative correlation and red indicating a stronger positive correlation (−1 < *r* < 1). (b) KEGG enrichment analysis of DEGs in the turquoise, blue and yellow modules. (c) Statistical analysis of transcription factor genes in the turquoise, blue and yellow modules.

WGCNA was conducted to identify transcription factors regulating SCs and NSCs synthesis in *PtrVINV2* transgenic plants, using fold changes of DEGs, SCs and NSCs contents and INV activity as the inputs. As shown in Figure 7a, WGCNA clustered the DEGs into 14 modules. The turquoise module was significantly positively correlated with lignin and cellulose contents (0.95 and 0.68, respectively), whereas the yellow module was significantly negatively correlated with lignin content (−0.9), and the magenta module was negatively correlated with cellulose content (−0.65). The yellow module exhibited a significant positive correlation with hemicellulose content (0.87), whereas the turquoise module exhibited a significant negative correlation (−0.87). The starch, sucrose contents and CWINV activity were significantly positively correlated with the red module (0.93, 0.9 and 0.88, respectively), and significantly negatively correlated with the blue module (−0.93, −0.91 and −0.88, respectively). The blue module was significantly positively correlated with glucose content and VINV activity (0.99 and 0.96, respectively), while the red module exhibited significant negative correlations (−0.98 and −0.96, respectively).

The blue, red, turquoise and yellow modules, which exhibited higher correlations with SC and NSC contents, were chosen for pathway annotation analysis, identification of transcription factor types and screening of ‘hub’ genes among their DEGs. The DEGs in these modules were enriched in pathways including MAPK signalling pathway‐plant, plant hormone signal transduction, amino sugar and nucleotide sugar metabolism, biosynthesis of secondary metabolites, carbon metabolism, starch and sucrose metabolism and phenylpropanoid biosynthesis (Figure 7b). Members of 22 transcription factor families were identified in the DEGs of the blue, red, turquoise and yellow modules, with *ERF*, *MYB* and *WRKY* being the most abundant (Figure 7c). In the blue module, the identified ‘hub’ transcription factor genes included *Potri.019G089000* (*bHLH*), *Potri.008G064200* (*MYB*) and *Potri.006G234300* (*C3H*), with *Potri.008G064200* potentially involved in xylem development (Petzold *et al*., 2018) (Table S1). The ‘hub’ transcription factor genes in the red module comprised the osmotic regulation‐related *Potri.001G219100* (*MYB*) (Song *et al*., 2019), the jasmonic acid signalling pathway participant *Potri.002G176900* (*bHLH*) (Ranjan *et al*., 2022) and the functionally unknown *Potri.001G323500* (*CO‐like*). The turquoise module included the ‘hub’ transcription factor *Potri.009G106600* (*G2‐like*), which is associated with abiotic stress (Wu *et al*., 2023) (Table S1). In the yellow module, the ‘hub’ transcription factor genes included *Potri.002G081000* (*NAC*), which is involved in plant salt tolerance mechanisms (Yao *et al*., 2019), and the functionally unknown *Potri.009G032900* (*GRAS*) gene (Table S1). Additionally, *PtrVINV2*, along with certain genes associated with cellulose, hemicellulose, lignin synthesis and hormone signalling pathways, were included in the blue, red, turquoise and yellow modules (Table S2). In conclusion, the overexpression or knockout of *PtrVINV2* was found to significantly alter the expression patterns of numerous transcription factor genes.

### Validation of transcriptomics data by RT‐qPCR

To affirm the accuracy of the transcriptome data, 10 genes from pathways related to starch and sucrose metabolic, amino sugars and nucleotide sugars metabolic, phenylpropane biosynthesis and plant hormone signal transduction were chosen for RT‐qPCR validation (Figure S6). The results demonstrated a significant correlation (*R* ^2^ = 0.9475) between the transcriptome and RT‐qPCR data, affirming the reliability of the transcriptome sequencing data.

### *PtrVINV2* knockout plants exhibit salt sensitivity

**Figure 8:**
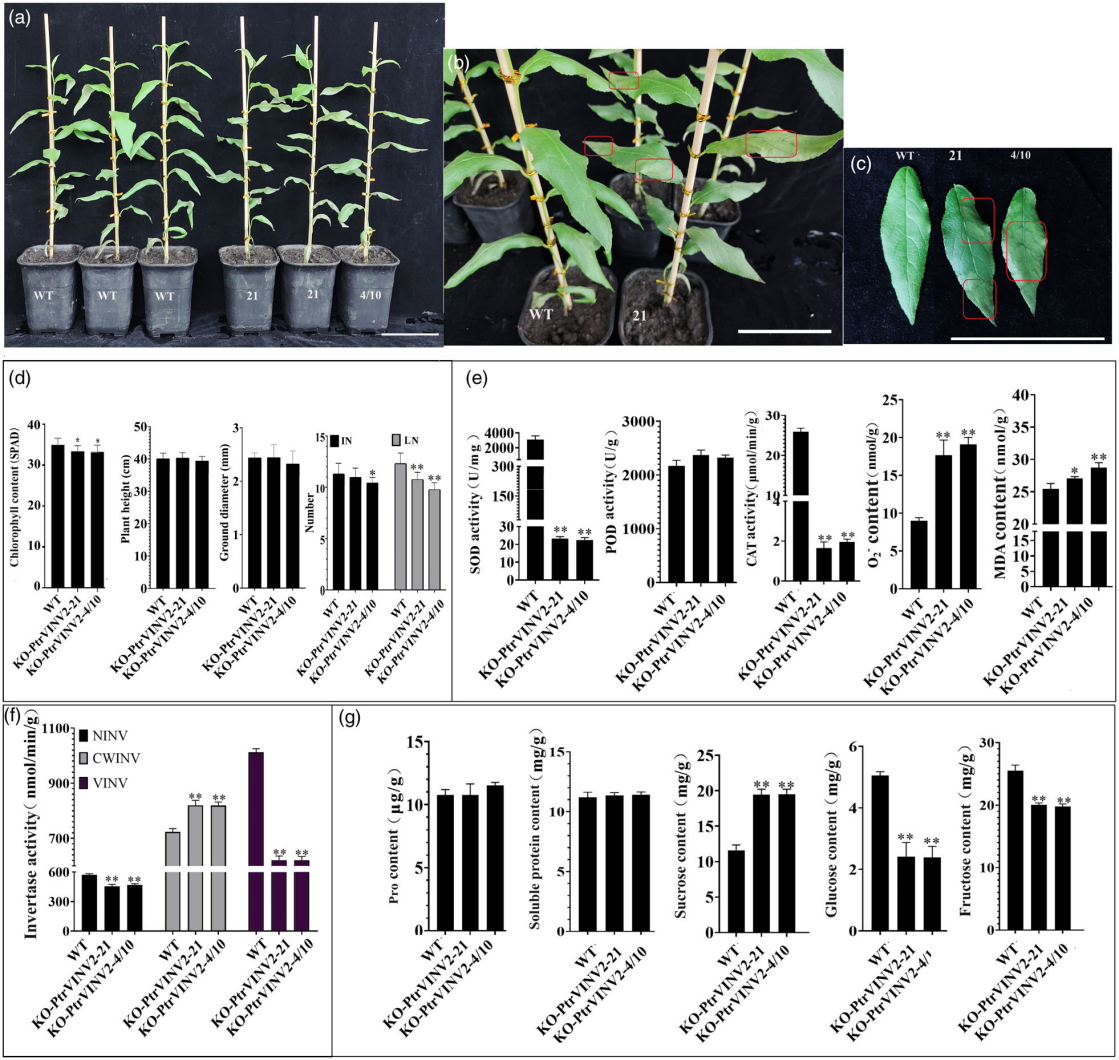
Phenotypic analysis of *PtrVINV2* knockout plants under salt treatment. (a) General growth condition of the plant. Bar = 10 cm. (b) Enlarged view of the entire plant. Bar = 10 cm. (c) Salt spots observed on the 4th leaf. Bar = 10 cm. The salt spots were highlighted using red boxes. (d) Growth indices, including chlorophyll content, plant height, ground diameter, number of leaves (LN) and number of internodes (IN). (e) Oxidative indices, including the activities of SOD, POD and CAT, as well as the contents of O₂^−^ and MDA. (f) Invertase activity. (g) Osmotic regulatory substances, including the contents of proline, sucrose, soluble protein, fructose and glucose. Asterisks indicate levels of statistical significance (*t*‐test; *P < 0.05, **P < 0.01).

The identification of transcription factor genes associated with salt tolerance as ‘hub’ genes within SCs and NSCs modules, combined with the role of VINV in regulating osmotic pressure, suggests that the *PtrVINV2* gene may participate in the salt tolerance mechanism. To investigate the response of the *PtrVINV2* gene to salt stress, the KO‐*PtrVINV2* lines were selected as treatment material due to the challenges associated with observing salt stress phenotypes in OE‐*PtrVINV2* (developing xylem‐specific promoter driver). Phenotypic observations revealed that, on the 6th day of treatment, distinct salt spots and signs of wilting were observed on the 4th leaf of the KO‐*PtrVINV2* lines (Figure 8a–c) and a significant decrease in chlorophyll content was observed (Figure 8d), while the 4th leaf of the WT maintained a healthy growth state, characterized by a deep green colour (Figure 8a–c). Furthermore, compared with WT, no significant differences were observed in plant height or ground diameter in KO‐*PtrVINV2* lines. However, leaf number was significantly reduced, and stem internode number was lower in KO‐*PtrVINV2* (Figure 8d). To investigate the possible reasons for the salt sensitivity of KO‐*PtrVINV2* lines, the contents of antioxidant indicators, osmotic regulatory substances and INV activity were assessed. The results indicated that under salt stress, the activities of SOD and CAT in KO‐*PtrVINV2* lines were significantly lower than those in WT, while the contents of O_2_^−^ and MDA were significantly elevated (Figure 8e). Additionally, under salt stress, the NINV and VINV were significantly reduced in KO‐*PtrVINV2* lines compared to the WT, while the CWINV was significantly increased (Figure 8f). Measurement of osmotic regulatory substances contents revealed that, compared to WT, the soluble protein and proline contents in KO‐*PtrVINV2* lines did not change significantly under salt stress. However, sucrose content was significantly elevated, while fructose and glucose levels were markedly reduced (Figure 8g). In summary, under salt stress, KO‐*PtrVINV2* lines displayed reduced contents of osmotic regulatory substances, diminished antioxidant capacity and increased cellular damage, and thus exhibited sensitivity to salt stress.

## Discussion

### *PtrVINV2* is not essential for cellulose synthesis

As society advances, reducing dependence on petroleum becomes an inevitable trend. Therefore, the utilization of renewable resources like lignocellulosic biomass for producing transportation fuels represents a novel approach (Ho *et al*., 2014; Mansfield *et al*., 2012). Poplar trees possess several characteristics that make them attractive as alternative sources for producing lignocellulosic biomass fuels. These include strong growth capabilities in nutrient‐poor soils, relatively high cellulose content (due to developed secondary growth), low ash and extractive contents and ease of biomass harvesting, processing and storage (Ragauskas *et al*., 2006). However, although poplar is an economically viable feedstock choice, there is a need to enhance its secondary growth capacity to increase lignocellulose production (Chudy *et al*., 2019; Shooshtarian *et al*., 2018). The secondary cell walls formed during secondary growth contain not only polysaccharides such as cellulose and hemicellulose but also structurally stable lignin, located on the outer side of cellulose and hemicellulose. Lignin typically restricts the release of accessible polysaccharides during enzymatic saccharification (Bryant *et al*., 2020). Consequently, efforts have been made to modify gene expression to alter cell wall characteristics, with the aim of reducing lignin recalcitrance (Bryant *et al*., 2020). Although the effects of genetic engineering on cell wall characteristics cannot be fully predicted, there is potential to generate transgenic poplar suitable for processing into biofuels. The carbon sources required for secondary growth are derived from sucrose produced via photosynthesis. Sucrose can be utilized by cells only after hydrolysis into hexoses. INVs, the key enzyme responsible for sucrose hydrolysis, plays a critical role in carbon utilization (Verbancic *et al*., 2018). Currently, NINV has been shown to provide carbon sources for SCs synthesis in poplar (Rende *et al*., 2017; Zhang *et al*., 2023). However, no reports have addressed whether acidic INVs in poplar are involved in the synthesis of SCs. In this study, the *PtrVINV2* gene was functionally characterized using genetic transformation. The findings revealed that knockout lines exhibited reduced lignin content and increased hemicellulose content, while cellulose levels remained unchanged. Unfortunately, the overexpression lines did not affect SCs content (Figure 5). These results suggest that the *PtrVINV2* gene is not essential for cellulose synthesis in *P. trichocarpa*. This may be attributed to the localization of proteins in the acidic INV subfamily to the cell wall and vacuole, whereas NINV family proteins are typically localized in the cytoplasm. Additionally, cellulose synthase complexes are situated at the plasma membrane, facing the cytoplasm to receive UDP‐glucose (Li *et al*., 2014; McFarlane *et al*., 2014). Thus, during the provision of carbon sources for cellulose synthesis, NINV family proteins may have a spatial advantage over acidic INV family proteins.

### *PtrVINV2* regulates secondary xylem development

The apical meristem in poplar produces procambium, which develops into primary vascular tissues (primary xylem and phloem) that dominate the early internodes (1–3 IN). Secondary growth begins at 3 IN and progresses through mid (4–5 IN) and late stages (beyond 6 IN), characterized by the transition to secondary xylem formation and lignified secondary wall structures (Constantinos *et al*., 2005; Dharmawardhana *et al*., 2010). In this study, a significant reduction in xylem width and cell layer number was observed in the KO‐*PtrVINV2* lines during the middle and late stages of xylem formation (Figure 3a,c,d), while fibre cell wall thickness remained unchanged (Figure 4a,c). This indicates that the reduced xylem width is primarily driven by fewer cell layers. Surprisingly, xylem width gradually normalized while the xylem cell layer still decreased with increasing secondary growth (Figure 3a,c,d). Several factors may contribute to this phenomenon. In knockout lines, reduced xylem cell numbers suggest impaired cambial cell proliferation; however, secondary growth compensates by expanding or differentiating existing xylem cells, maintaining xylem width (Zhang *et al*., 2024). Xylem function may be stabilized through compensatory mechanisms, such as increasing individual xylem cell size or altering morphology, resulting in normal xylem width despite fewer cell layers (Ben‐Targem *et al*., 2021). The initial reduction in xylem cell layers may indicate a delay in early xylem development, but secondary growth may partially restore xylem width through the preferential differentiation of vessels or fibres, which are critical for radial expansion (Cornelis and Hazak, 2022). Genetic redundancy and plasticity in plant networks may compensate for lost function in knockout lines, contributing to the normalization of xylem width over time, even if the xylem cell layers do not fully recover (Cornelis and Hazak, 2022). Furthermore, the xylem width in the OE‐*PtrVINV2* lines increased significantly, despite an unchanged number of cell layers (Figure 3). This increase was accompanied by thicker fibre cell walls (Figure 4), suggesting that fibre cell wall thickness, rather than cell layer number, is the main contributor to increased xylem width in the overexpression lines. The distinct xylem phenotypes in overexpression and knockout plants arise from different underlying causes, likely resulting from the specific activation of gene expression by the DX15 promoter in the overexpression lines. The larger stem diameter in the KO‐*PtrVINV2* lines, compared to the WT, may be attributed to a significant increase in pith diameter in the knockout lines.

### *PtrVINV2* regulates lignin synthesis through sugar and hormonal signals

Plant hormones are known to regulate the lignin biosynthetic pathway. Auxin directly regulates lignin accumulation (Cancé *et al*., 2022; Ghelli *et al*., 2018; Qu *et al*., 2021; Wang *et al*., 2024; Xu *et al*., 2023a). For instance, in *P. edulis*, *ARF3* and *ARF6* are transcriptional activators that bind directly to the promoter regions of genes encoding key enzymes in the lignin biosynthetic pathway, such as 4‐coumarate‐CoA ligase (*4CL3*, *4CL7* and *4CL9*) and caffeoyl‐CoA O‐methyltransferase (*CCoAOMT2*), thereby activating their expression. The ability of this binding depends on auxin concentration (Wang *et al*., 2024). Gibberellins promote stem elongation and wood formation (Biemelt *et al*., 2004; Eriksson *et al*., 2000). For instance, transgenic poplar lines expressing *PdGA20ox1*, which encodes an enzyme producing bioactive gibberellins, and *PtrMYB221*, which encodes a MYB transcription factor that negatively regulates lignin biosynthesis, have exhibited reduced lignin content, significantly improved saccharification efficiency and increased cellulose content (Cho *et al*., 2019). Jasmonic acid also regulates lignin synthesis pathways. For instance, the overexpression of the jasmonic acid‐induced *R2R3 MYB‐type* transcription factor gene (*NtMYBJS1*), which is tightly co‐expressed with *PAL* and *4CL*, resulted in the specific accumulation of phenylpropanoid compounds in tobacco BY‐2 cells (Galis *et al*., 2006). Sucrose metabolism also functions as a signalling mechanism in plant development, correlating with plant hormone signalling pathways. For instance, in *Arabidopsis*, glucose signalling is known to coordinate auxin biosynthesis and signalling (Mishra *et al*., 2009) and cytokinin (Kushwah and Laxmi, 2017). In this study, the expression profiles of genes related to the lignin synthesis pathway (Figure 6b) and plant hormone signalling pathways (Figure 6c) were altered in *PtrVINV2* transgenic plants. In plants, SCs content typically exhibits a certain level of stability. Given that the hemicellulose content is altered following *PtrVINV2* transformation, plants may regulate the lignin synthesis pathway to maintain SCs content. Therefore, it is hypothesized that overexpression or knockout of the *PtrVINV2* gene alters the levels of sucrose, glucose and fructose, which affects their roles as signalling molecules in regulating plant hormone signalling, thereby modulating the expression of genes involved in lignin synthesis to maintain stable SCs content.

### Overview of differentially expressed transcription factors in *PtrVINV2* transgenic plants

Wood formation is regulated by a transcriptional network, with some transcription factors identified as marker genes for wood development. For example, studies on protein–protein and protein‐DNA interactions in the wood development of *P. trichocarpa* have demonstrated that Potri.008G064200, a member of the MYB transcription factor family, functions as a key regulator in the wood formation network (Petzold *et al*., 2018). Overexpressing *PtrbHLH186* (*Potri.018G083700*) in *P. trichocarpa* resulted in retarded plant growth, increased guaiacyl lignin, a higher proportion of smaller stem vessels and strong drought‐tolerant phenotypes (Liu *et al*., 2022). Overexpression of *PtVNS07/PtrWND6A* (*Potri.013G113100*) induced ectopic secondary wall thickening in poplar as well as in *Arabidopsis* seedlings (Ohtani *et al*., 2011). In this study, *Potri.008G064200* acts as a ‘hub’ transcription factor gene within the SCs/NSCs modules of *PtrVINV2* transgenic plants. Although *PtrbHLH186* and *PtrWND6A* are not hub transcription factor genes in the SCs/NSCs modules, *PtrbHLH186* was significantly downregulated in the KOV vs. WT group (Figure S7), accompanied by a significant reduction in lignin content in the KO‐*PtrVINV2* lines (Figure 5b). *PtrWND6A* was significantly upregulated in the OEV vs. WT group (Figure S7), with a corresponding increase in fibre cell wall thickness in the OE‐*PtrVINV2* lines (Figure 4a,c). Therefore, further functional analysis of *Potri.008G064200*, *Potri.018G083700*, *Potri.013G113100* is necessary to clarify their roles in wood formation in *P. trichocarpa* and to link their functions to the secondary growth phenotypes observed in *PtrVINV2* transgenic lines. This investigation may contribute to a deeper understanding of the regulatory network governing wood development.

Furthermore, 209 differentially regulated osmotic response promoters were identified in *Populus simonii* Carr., with 26% linked to the interaction of Potri.001G219100 in the regulation of salt tolerance processes (Song *et al*., 2019). Potri.009G106600 is classified within the G2‐like (GLK) transcription factor family of *P. trichocarpa*, responsible for regulating chloroplast development. Most members of this family are implicated in responses to abiotic stresses, including cold and osmotic stress, as well as plant hormones such as MeJA and GA (Wu *et al*., 2023). In *Populus simonii* Carr. × *Populus nigra* L., transcriptomic analyses revealed that Potri.002G081000 from the NAC family exerts a synergistic effect in enhancing the salt tolerance of transgenic poplar lines overexpressing *ERF76* (Yao *et al*., 2019). In this study, *Potri.001G219100*, *Potri.009G106600* and *Potri.002G081000* were identified as ‘hub’ genes within the SCs/NSCs modules of *PtrVINV2* transgenic plants (Table S1). It is hypothesized that these three transcription factors regulate salt tolerance mechanisms in *PtrVINV2* transgenic plants.

### *PtrVINV2* gene involvement in plant salt tolerance

Soil salinization is a major environmental challenge, with salt‐affected land increasing by 10% annually (Hnilickova *et al*., 2021). Plants adapt to salinization through mechanisms like osmotic regulation, ion homeostasis and antioxidant/hormonal responses (Boriboonkaset *et al*., 2013). Osmotic adjustment involves accumulating substances such as sugars, polyols, proline and glycine betaine to mitigate salt stress (Ashraf and Foolad, 2007; Gupta and Kaur, 2005; Stoop *et al*., 1996). The accumulation of sugars in plant cells is among the most effective mechanisms through which osmotic solutes act as osmotic regulators in salt defence (Cha‐Um *et al*., 2009; Gao *et al*., 1998; Gibson, 2000; Olmos and Hellin, 1996). INVs catalyse the hydrolysis of sucrose to generate glucose and fructose, which play critical roles in sugar accumulation and osmotic pressure regulation (Ruan *et al*., 2010). Although INVs' role in salt resistance is established in some grasses, data on woody plants are lacking (Cheng *et al*., 2023; Mao *et al*., 2024). In this study, several ‘hub’ transcription factors related to salt tolerance were identified in the SCs/NSCs modules, while KO‐*PtrVINV2* plants exhibited salt sensitivity under high salinity stress, with reduced VINV activity, increased sucrose content and decreased fructose and glucose levels (Figures 7 and 8). The salt sensitivity of KO‐*PtrVINV2* plants is likely attributed to reduced sucrose hydrolysis and limited osmotic regulator synthesis, compounded by decreased antioxidant capacity, resulting in increased cellular damage and heightened salt sensitivity (Figure 8). These findings provide excellent candidate genes for the breeding of salt‐tolerant forest tree varieties.

### Role of *PtrVINV2* gene in lignin and hemicellulose synthesis

VINV catalyses the hydrolysis of sucrose to produce glucose and fructose, which serve not only as sugar signals but also as substrates in the synthesis of polysaccharides, including cellulose and hemicellulose, through several catalytic steps (Mishra *et al*., 2009; Verbancic *et al*., 2018). In this study, the expression pattern of *PtrVINV2* was positively correlated with the expression of *SUT*, *SUS* and *ADP‐Glc PPase* genes while being negatively correlated with the expression of *CWINV*, *STP*, *AXS*, *UAM*, *GATL* and *GAUT/PAE* genes, as well as with hemicellulose content. Moreover, PtrVINV2 expression was positively correlated with genes involved in plant hormone signalling pathways, including those related to cytokinin and salicylic acid, as well as genes associated with lignin biosynthesis, such as HCT, COMT, CAD and Peroxidase, and with lignin content (Figures 5 and 6). Previous studies have demonstrated that plant hormones and sugar signalling pathways regulate lignin content (Cancé *et al*., 2022; Mishra *et al*., 2009). Based on the above results, it is hypothesized that the *PtrVINV2* gene facilitates the transport of sucrose in the apoplast into sink cells via SUT. However, this sucrose is likely not directly utilized in cellulose synthesis. Instead, it likely serves as a sugar signal to activate plant hormone signalling pathways, upregulating genes associated with lignin biosynthesis and promoting lignin synthesis, while reducing hemicellulose accumulation. In summary, the *PtrVINV2* gene regulates lignin and hemicellulose synthesis, promoting secondary xylem development.

### Relationship between cell wall components and salt stress in *PtrVINV2* transgenic plants

A key adaptation of plants to salt stress involves the regulation of growth, accompanied by dynamic changes and rearrangements in the cell wall (Cosgrove, 2015; Tenhaken, 2014; Wang *et al*., 2016). The plant cell wall is a dynamic structure, with cellulose microfibrils embedded in an amorphous matrix composed of pectin, hemicellulose polysaccharides, structural proteins and lignin (Herburger *et al*., 2020; Mutwil *et al*., 2008; Scheller and Ulvskov, 2010). The synthesis of cell wall polysaccharides plays a crucial role in the plant's response to salt stress. For instance, knockout of *CesA6* leads to salt sensitivity (Zhang *et al*., 2016), and in *Arabidopsis*, increased accumulation of β‐1,4‐galactan heightens salt stress sensitivity (Yan *et al*., 2021). Furthermore, the glycosyl isomerase UGE3 has been shown to enhance mechanical strength and salt tolerance in rice by mediating cell wall polysaccharide accumulation (Tang *et al*., 2022). In this study, knockout plants exhibited a reduced sucrose metabolism capacity, along with significant changes in lignin and hemicellulose content (Figures 2 and 5), suggesting alterations in cell wall mechanical strength. Additionally, these knockout plants showed increased sensitivity to salt stress (Figure 8). Based on these findings and existing literature, it is hypothesized that PtrVINV2 regulates mechanical strength by modifying the composition of cell wall components, specifically lignin and hemicellulose, thereby improving plant tolerance to salt stress.

## Materials and methods

### Plant material and growth conditions

The *P. trichocarpa* genotype Nisqually‐1, cultivated in the greenhouse of the State Key Laboratory of Tree Genetics and Breeding in Northeast Forestry University, was utilized as the experimental material in this study. Sterile plantlets were propagated in a growth chamber (25–27°C, 16 h : 8 h, light : dark photoperiod) under a light intensity of 60–80 μmol/m^2^/s for genetic transformation. Shoot apices and lateral buds from transgenic young trees were excised, rooted in water for 3 weeks and subsequently transplanted into humus soil for asexual propagation (Xu *et al*., 2021). The generated plantlets were cultivated in a glasshouse (25–28°C, 16 h : 8 h, light : dark photoperiod) under a light intensity of 250 μmol/m^2^/s for phenotypic analysis. Plant height, stem diameter (the 4th internode, 4 IN; the 8th internode, 8 IN; the 16th internode, 16 IN; and ground diameter, GD), leaf count and internode count of transgenic plants were measured and recorded using a ruler and vernier calliper. Three biological replicates were established for each line, with measurements taken from nine plants per replicate.

### Subcellular localization analysis

The full‐length sequence of the *PtrVINV2* gene (*Potri.003G112600*) was cloned using polymerase chain reaction (PCR) technology, with complementary DNA (cDNA) from WT serving as the template. A subcellular localization vector was constructed based on the pBS‐35S::GFP vector. Recombinant vectors (pBS‐35S::PtrVINV2‐GFP), negative control vectors (pBS‐35S::GFP) and positive control vectors (Vac‐rk CD3‐975, a vacuolar membrane protein marker) (Nelson *et al*., 2007) were used to bombard onion epidermal cells. Fluorescence was then observed using laser confocal microscopy (LSM 900, Oberkochen, Germany). Plasmolysis was then performed with a 0.3 mg/mL sucrose solution during microscopic observation. The primers used in this study are listed in Table S3.

### Vector construction and genetic transformation

The promoter sequence of the *Potri.009G012200* (DX15, developing xylem‐specific expression) was cloned using PCR with total DNA as the template. Subsequently, the DX15 promoter and the full‐length *PtrVINV2* sequence were used to replace the 35S promoter in the pROKII vector through restriction enzyme digestion and ligation techniques to construct the vector of overexpression in developing xylem. To construct the CRISPR/Cas9 vector, candidate target sites were initially screened on the first exon of the *PtrVINV2* gene using the CRISPR‐GE website (http://skl.scau.edu.cn/, last accessed July 7, 2024). To further confirm the specificity of the target sequences, the candidate sites were subjected to BLAST analysis in the *P. trichocarpa* genome database (https://phytozome‐next.jgi.doe.gov/blast‐search, last accessed July 7, 2024). The knockout vector of the *PtrVINV2* gene was subsequently constructed using the Golden Gate method. PCR fragments were amplified using pCBC‐DT1T2 as a template, then purified and ligated into the pKSE401 plasmid using BsaI and T_4_ ligase (Xing *et al*., 2014). The recombinant overexpression and knockout vectors were transformed into the *Agrobacterium* GV3101 strain, followed by the genetic transformation of *P. trichocarpa* as described by Li *et al*. (2017). Plants that survived in the culture medium containing 50 mmol/L kanamycin were designated as resistant plants.

### Overexpression and knockout plant identification

Total DNA and RNA of overexpressing‐resistant plants were extracted using the CTAB method (Gambino *et al*., 2008). The total DNA was subsequently used as a template for PCR to verify the presence of the exogenous *PtrVINV2* gene, using primers specific to *PtrVINV2* (with upstream and downstream primers designed from separate exons). The cDNA synthesized from RNA then was used as a template to quantify the transcription abundance of the *PtrVINV2* gene through qRT‐PCR technology. Additionally, knockout plants were identified through PCR verification using zCas9‐specific primers to confirm the integration of the CRISPR/Cas9 vector's T‐DNA into the genome of resistant lines. Subsequently, the total DNA from transgenic plants with integrated T‐DNA was used as a template to amplify target sequences using PCR. The PCR products were then sequenced at the Hi‐TOM platform of the China National Rice Research Institute, Chinese Academy of Agricultural Sciences (http://121.40.237.174/Hi‐TOM/Sample_acceptance_select_sanyang.php, last accessed July 7, 2024), with each sample subjected to 2000 reads and a filtering threshold of 1% to identify the editing types of knockout plants. The *PtrVINV2* gene overexpression and knockout plants were labelled as OE‐*PtrVINV2*‐line and KO‐*PtrVINV2*‐line, respectively.

### Enzyme activity assays

The bark tissue was excised from the stems of 3‐month‐old transgenic plants, and the developing xylem was carefully scraped using a surgical blade for invertase activity assays. The activities of NINV, CWINV and VINV were measured following previously established protocols (Rende *et al*., 2017; Samac *et al*., 2015).

### Non‐structural carbohydrate and structural carbohydrate contents assays

In plants, carbon is classified into two categories: SCs and NSCs. SCs are found in an inactive state and serve as the primary substance in plant cell morphogenesis. It primarily consists of various macromolecular compounds, including lignin, cellulose, hemicellulose and pectin. NSCs are maintained in an active state and serve as a key energy transport substance during plant growth. It primarily consists of soluble sugars, including monosaccharides like glucose and fructose, disaccharides such as sucrose and maltose, and polysaccharides such as starch (Raessler *et al*., 2010). Therefore, in this study, the quantities of major SCs components—lignin, cellulose and hemicellulose—were measured to reflect alterations in SCs content. Stems from three‐month‐old transgenic plants were initially peeled, dried and ground into a fine powder. The powder was subsequently washed with 70% ethanol, chloroform/methanol (1 : 1 v/v) and acetone to yield insoluble samples. These samples were analysed to determine cellulose and lignin content. Lignin content was quantified using the acetyl bromide spectrophotometric method (Foster *et al*., 2010a). Cellulose content was measured according to established methods (Foster *et al*., 2010b). Hemicellulose content was determined using a hemicellulose content assay kit (Suzhou Kming Biotechnology Co., Ltd., Suzhou, China). Additionally, the major components of NSCs—starch, sucrose, glucose and fructose—were quantified to evaluate changes in NSCs content. Starch, sucrose, glucose and fructose contents were measured using appropriate assay kits (Suzhou Kming Biotechnology Co., Ltd.).

### Microstructure analysis

To identify phenotypic changes during the early, middle and late stages of wood formation in transgenic plants, paraffin sections were prepared from the 2nd, 4th, 6th, 8th and 10th stem internodes of three‐month‐old plants (Constantinos *et al*., 2005; Dharmawardhana *et al*., 2010). The development of xylem was observed using toluidine blue and phloroglucinol‐HCl staining (Liu *et al*., 2015). To further assess changes in fibre cells, free‐hand cross‐sections were performed on the 10th internode of three‐month‐old plants. The fresh sections were gold‐coated and transferred to a benchtop scanning electron microscope (JCM‐5000, Tokyo, Japan) for observation and imaging. Additionally, the 10th internode was sectioned into 1 cm segments using a double‐edged blade. After bark removal, the segments were immersed in a delignification solution (1 : 1 v/v nitric acid‐chromic acid). After incubation at 60°C for 1–2 h, the samples were rinsed with ultrapure water and stored. Fibre cell dimensions were examined under an optical microscope (Zeiss, Oberkochen, Germany) after staining with 1% acid fuchsin solution. The xylem width, fibre cell wall thickness, fibre cell length and width, number of fibre cells and number of vessel elements were quantified using ImageJ software. Three biological replicates and three technical replicates were performed.

### Transcriptome analysis

Total RNA was extracted from the developing xylem of overexpression, knockout and WT plants using the CTAB method (Gambino *et al*., 2008). The NEBNext Ultra RNA Library Prep Kit for Illumina (California, USA) was used for library construction and sequencing was performed on an Illumina HiSeq platform at ANOROAD (Beijing, China). The paired‐end reads were aligned to the reference genome (http://ftp.ensemblgenomes.org/pub/plants/release‐52/fasta/populus_trichocarpa/dna/, accessed July 7, 2024) using HISAT2 software (version 2.2.1). Gene expression levels were quantified using the fragments per kilobase of transcript per million mapped reads (FPKM) method. Differentially expressed genes (DEGs) were identified using DESeq2 (version 1.22.1). The false discovery rate (FDR) was calculated by applying the Benjamini–Hochberg correction method to the hypothesis test *P*‐values after differential analysis. DEGs were selected based on the thresholds of fold change ≥1.5 and FDR <0.05. For KEGG, heatmaps, volcano plots and the weighted gene co‐expression network analysis (WGCNA) were performed using R software (version: 4.3.2, www.r‐project.org/, last accessed July 7, 2024).

### Salt treatment and phenotypic analysis

Knockout lines were clonally propagated according to the method described by Xu *et al*. (2021). The WT and knockout line seedlings were sown in a substrate composed of peat soil and vermiculite (V : V = 2 : 1) and cultured in a greenhouse (25–28°C, 16 h : 8 h, light : dark photoperiod). After 4 weeks of growth, healthy WT and knockout lines seedlings of the same size were selected and treated with 200 mL NaCl (200 mmol/L) for 10 consecutive days, with 200 mM NaCl applied every 2 days, while phenotypic changes were monitored. The 4th to 6th fully expanded leaves from the top were then collected and ground in liquid nitrogen for physiological analysis. In addition, chlorophyll content in the 4th fully expanded leaf from the top was determined using a chlorophyll meter (TYS‐4N, Jinkeliada Co., Shandong, China). For each line, 9 plants were measured, with 3 readings taken per plant. The content of SS, Pro, superoxide anion (O₂^−^), malondialdehyde (MDA), as well as the activity of POD, SOD, CAT were assessed using assay kits (Suzhou Kming Biotechnology Co., LTD., Suzhou, China).

#### Data analysis

SPSS 22.0 software (SPSS Inc., Chicago, IL, USA) was used to conduct a one‐way ANOVA and Duncan's test for the data. The figures presented in this study were generated using the software of GraphPad Prism (version 9.0).

## Conflict of interest

The authors declare no competing financial interests.

## Author contributions

S.Z.: writing—original draft and investigation; L.C.: investigation and software; D.C. and R.C.: data curation; methodology; J.C.: investigation; Q.Z.: validation; Y.Q.: methodology; G.L.: writing—review and editing; Z.X.: conceptualization and project administration.

## Supporting information


**Figure S1** Correlation analyses between contents of total nitrogen, soluble sugars, reducing sugars, starch, lignin, cellulose and hemicellulose, and transcript levels of *PtrVINVs* in different groups. In maps, the scale bar is on the right, and different colours of the cells indicate the degree of correlation. Asterisk (*) indicates a significant correlation. Data are from a previous study (Zhang *et al*., 2023).

**Figure S2** Stem growth trait analysis of *PtrVINV2* transgenic lines. (A) Growth status of plants, scale = 10 cm. (B) Statistical analysis of plant height. (C) Statistical analysis of leaf number and internode number, where L represents leaf number and IN represents internode number. (D) Statistical analysis of internode diameter, where 4 IN, 8 IN and 16 IN represent the 4th, 8th and 16th internodes, respectively, and GD represents ground diameter. (E) Statistical analysis of internode length. Asterisks indicate significant differences (*t*‐test, *P < 0.05, **P < 0.01).

**Figure S3** Microscopic images of fibre cells and vessel elements from the same area of the 10th internode in *PtrVINV2* transgenic lines. Scale bars = 200 μm.

**Figure S4** Assessment of transcriptomic sample quality. (A) Principal component analysis of samples, X axis represents the first principal component, Y axis represents the second principal component. (B) Hierarchical clustering heatmap analysis of samples. (C) Sample correlation analysis.

**Figure S5** Identification and functional classification of differentially expressed genes (DEGs). (A) Identification of DEGs in different comparison groups. (B) KEGG enrichment analysis. Pathways of interest are marked with asterisks: pathways jointly enriched in DEGs in knockout and overexpression plants are highlighted in red, and pathways enriched with DEGs exclusively in either overexpression or knockout plants are highlighted in green.

**Figure S6** Scatter plot showing a correlation between gene transcript levels estimated from transcriptome data and those determined by RT‐qPCR.

**Figure S7** Expression patterns of *PtrbHLH186*, and *PtrWND6A* genes in *PtrVINV2* transgenic plants. OEV and KOV refer to the *PtrVINV2‐*overexpression and ‐knockout lines, respectively.

**Table S1** Connectivity of ‘hub’ transcription factors in the blue, red, turquoise and yellow modules.

**Table S2** Network connectivity of the *PtrVINV2* gene, along with certain genes associated with cellulose, hemicellulose, lignin synthesis and hormone signalling pathways in blue, red, turquoise and yellow modules.

**Table S3** Sequences of all primers used in this study.

## Data Availability

The data that supports the findings of this study are available in the supplementary material of this article.
