# Supplementary material for: PtrVINV2 is dispensable for cellulose synthesis but essential for salt tolerance in Populus trichocarpa Torr. and Gray
Source: Plant Biotechnol J. 2025 Feb 24;23(6):1892–908. doi: 10.1111/pbi.70022 (PMC12120930; doi:10.1111/pbi.70022)
Supplement: Supplementary file 1 — Figure S1 Correlation analyses between contents of total nitrogen, soluble sugars, reducing sugars, starch, lignin, cellulose and hemicellulose, and transcript levels of PtrVINVs in different groups. In maps, the scale bar is on the right, and different colours of the cells indicate the degree of correlation. Asterisk (*) indicates a significant correlation. Data are from a previous study (Zhang et al., 2023). [file PBI-23-1892-s004.docx]

| 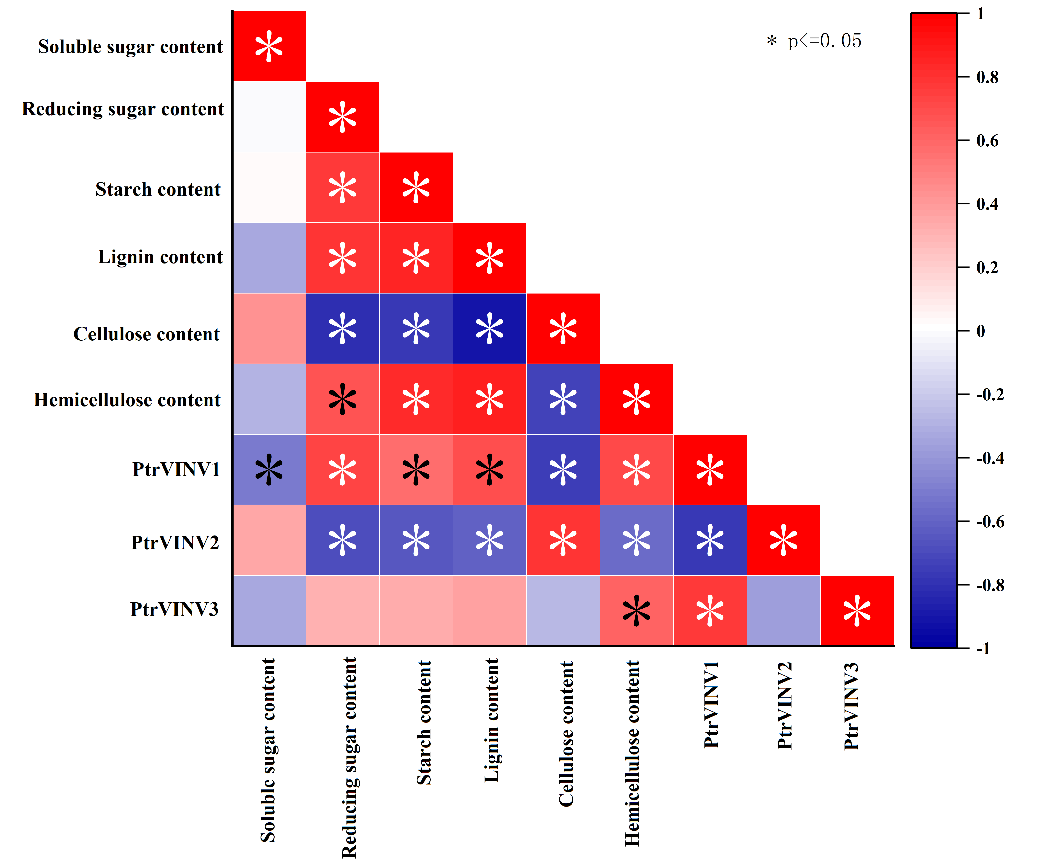 |
| --- |

**Figure S1** Correlation analyses between contents of total nitrogen, soluble sugars, reducing sugars, starch, lignin, cellulose, and hemicellulose, and transcript levels of *PtrVINVs* in different groups. In maps, scale bar is on the right, and different colors of the cells indicate degree of correlation. Asterisk (*) indicates a significant correlation. Data are from a previous study (Zhang et al., 2023).
