# Supplementary material for: PtrVINV2 is dispensable for cellulose synthesis but essential for salt tolerance in Populus trichocarpa Torr. and Gray
Source: Plant Biotechnol J. 2025 Feb 24;23(6):1892–908. doi: 10.1111/pbi.70022 (PMC12120930; doi:10.1111/pbi.70022)
Supplement: Supplementary file 2 — Figure S2 Stem growth trait analysis of PtrVINV2 transgenic lines. (A) Growth status of plants, scale = 10 cm. (B) Statistical analysis of plant height. (C) Statistical analysis of leaf number and internode number, where L represents leaf number and IN represents internode number. (D) Statistical analysis of internode diameter, where 4 IN, 8 IN and 16 IN represent the 4th, 8th and 16th internodes, respectively, and GD represents ground diameter. (E) Statistical analysis of internode length. Asterisks indicate significant differences (t‐test, *P < 0.05, **P < 0.01). [file PBI-23-1892-s005.docx]

| 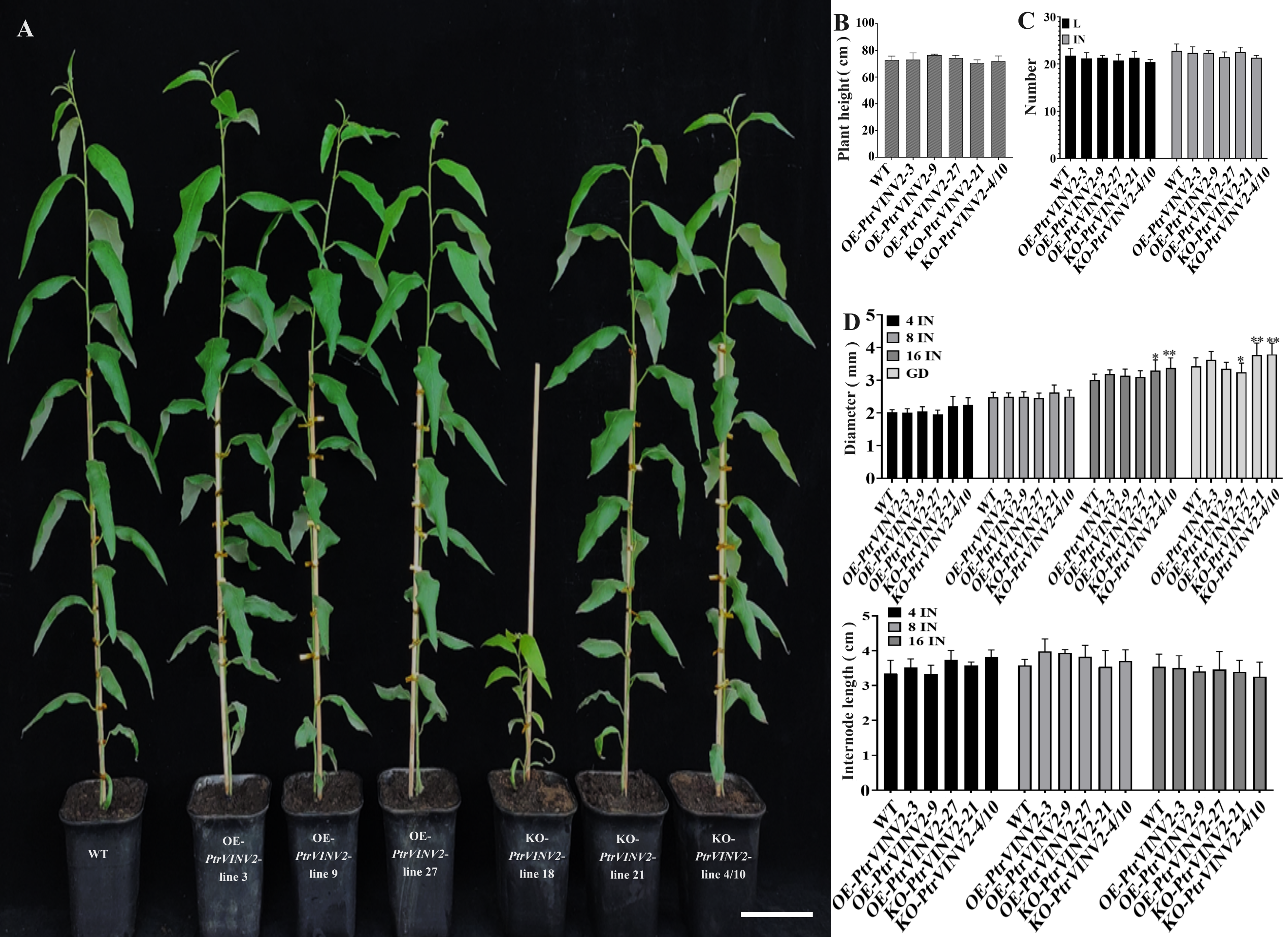 |
| --- |

**Figure S2** Stem growth trait analysis of *PtrVINV2* transgenic lines. (A) Growth status of plants, scale = 10 cm. (B) Statistical analysis of plant height. (C) Statistical analysis of leaf number and internode number, where L represents leaf number and IN represents internode number. (D) Statistical analysis of internode diameter, where 4 IN, 8 IN, 16 IN represent the 4^th^, 8^th^, and 16^th^ internodes, respectively, and GD represents ground diameter. (E) Statistical analysis of internode length. Asterisks indicate significant differences (*t*-test, * *p* < 0.05, ** *p* < 0.01).
