# Supplementary material for: PtrVINV2 is dispensable for cellulose synthesis but essential for salt tolerance in Populus trichocarpa Torr. and Gray
Source: Plant Biotechnol J. 2025 Feb 24;23(6):1892–908. doi: 10.1111/pbi.70022 (PMC12120930; doi:10.1111/pbi.70022)
Supplement: Supplementary file 3 — Figure S3 Microscopic images of fibre cells and vessel elements from the same area of the 10th internode in PtrVINV2 transgenic lines. Scale bars = 200 μm. [file PBI-23-1892-s001.docx]

| 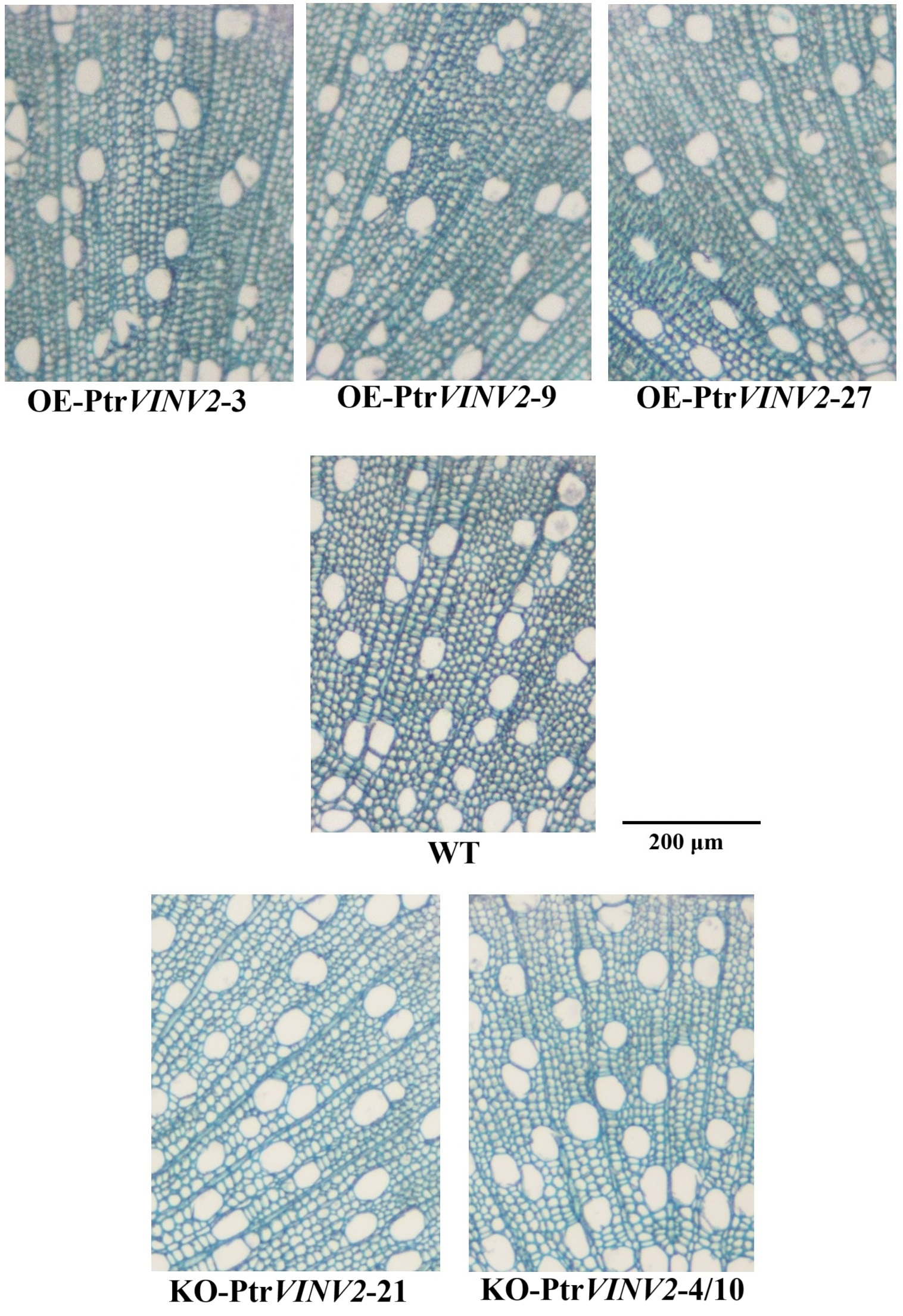 |
| --- |

**Figure S3** Microscopic images of fiber cells and vessel elements from the same area of the 10th internode in *PtrVINV2* transgenic lines. Scale bars = 200 μm.
