# Supplementary material for: PtrVINV2 is dispensable for cellulose synthesis but essential for salt tolerance in Populus trichocarpa Torr. and Gray
Source: Plant Biotechnol J. 2025 Feb 24;23(6):1892–908. doi: 10.1111/pbi.70022 (PMC12120930; doi:10.1111/pbi.70022)
Supplement: Supplementary file 4 — Figure S4 Assessment of transcriptomic sample quality. (A) Principal component analysis of samples, X axis represents the first principal component, Y axis represents the second principal component. (B) Hierarchical clustering heatmap analysis of samples. (C) Sample correlation analysis. [file PBI-23-1892-s007.docx]

| 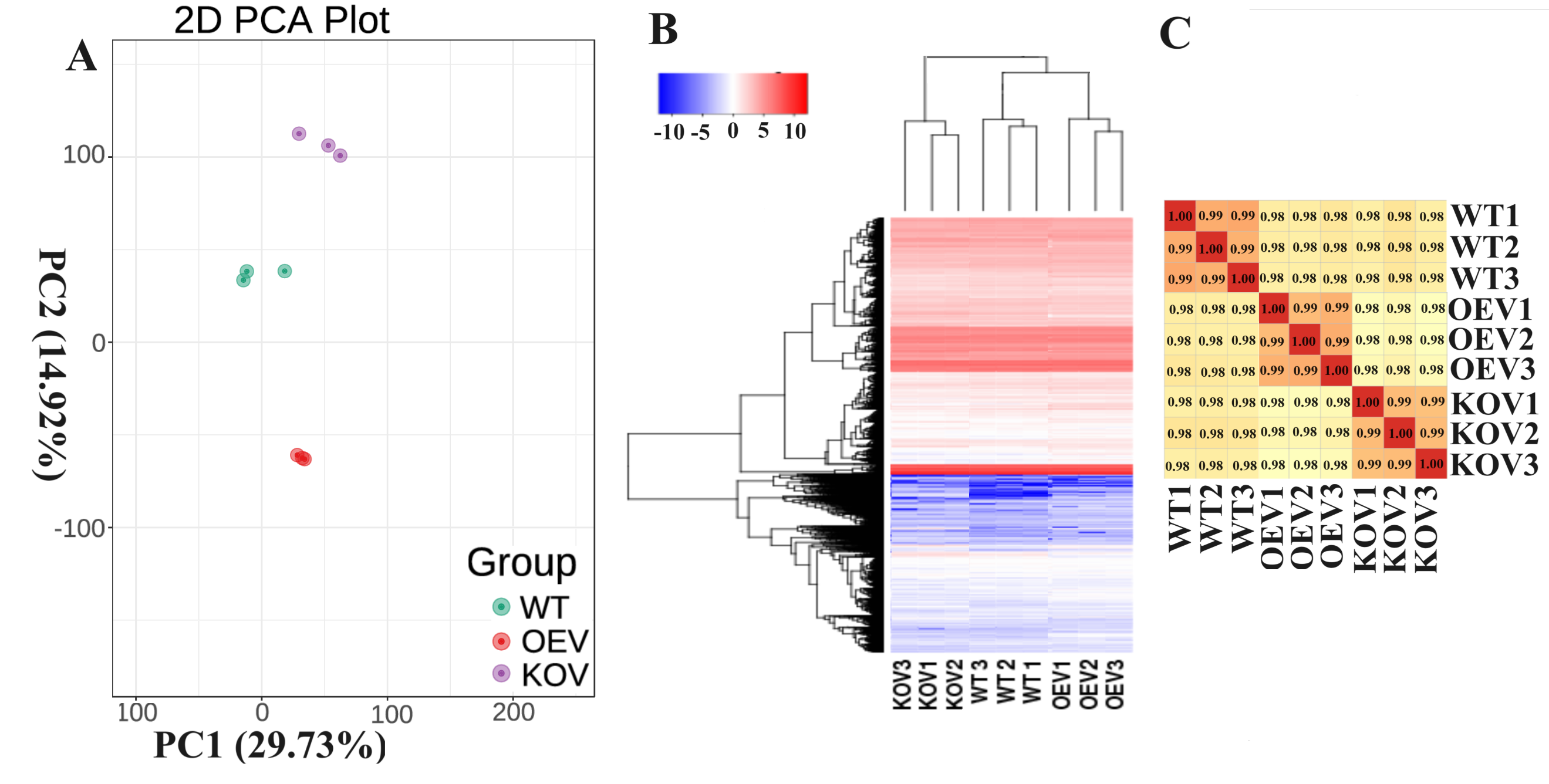 |
| --- |

**Figure S4** Assessment of transcriptomic sample quality. (A) Principal component analysis of samples, X-axis represents first principal component, Y-axis represents second principal component. (B) Hierarchical clustering heatmap analysis of samples. (C) Sample correlation analysis.
