# Supplementary material for: PtrVINV2 is dispensable for cellulose synthesis but essential for salt tolerance in Populus trichocarpa Torr. and Gray
Source: Plant Biotechnol J. 2025 Feb 24;23(6):1892–908. doi: 10.1111/pbi.70022 (PMC12120930; doi:10.1111/pbi.70022)
Supplement: Supplementary file 5 — Figure S5 Identification and functional classification of differentially expressed genes (DEGs). (A) Identification of DEGs in different comparison groups. (B) KEGG enrichment analysis. Pathways of interest are marked with asterisks: pathways jointly enriched in DEGs in knockout and overexpression plants are highlighted in red, and pathways enriched with DEGs exclusively in either overexpression or knockout plants are highlighted in green. [file PBI-23-1892-s009.docx]

| 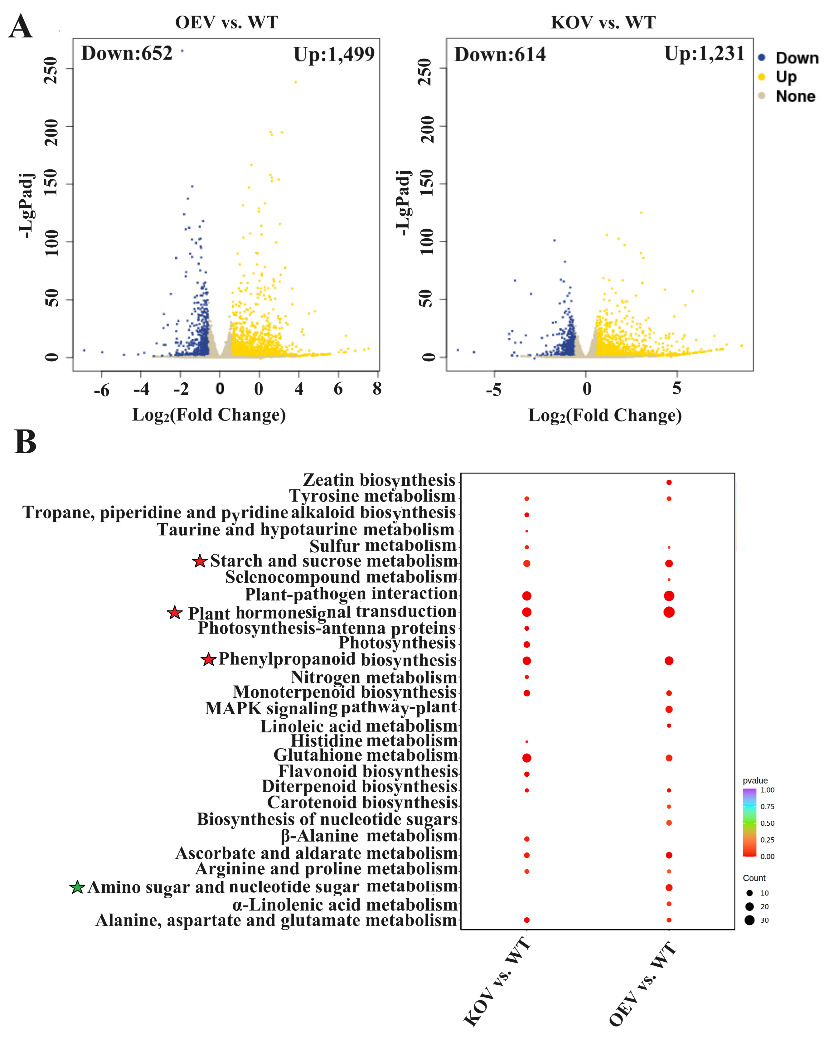 |
| --- |

**Figure S5** Identification and functional classification of differentially expressed genes (DEGs). (A) Identification of DEGs in different comparison groups. (B) KEGG enrichment analysis. Pathways of interest are marked with asterisks: pathways jointly enriched in DEGs in knockout and overexpression plants are highlighted in red, pathways enriched with DEGs exclusively in either overexpression or knockout plants are highlighted in green.
