# Supplementary material for: PtrVINV2 is dispensable for cellulose synthesis but essential for salt tolerance in Populus trichocarpa Torr. and Gray
Source: Plant Biotechnol J. 2025 Feb 24;23(6):1892–908. doi: 10.1111/pbi.70022 (PMC12120930; doi:10.1111/pbi.70022)
Supplement: Supplementary file 6 — Figure S6 Scatter plot showing a correlation between gene transcript levels estimated from transcriptome data and those determined by RT‐qPCR. [file PBI-23-1892-s010.docx]

| 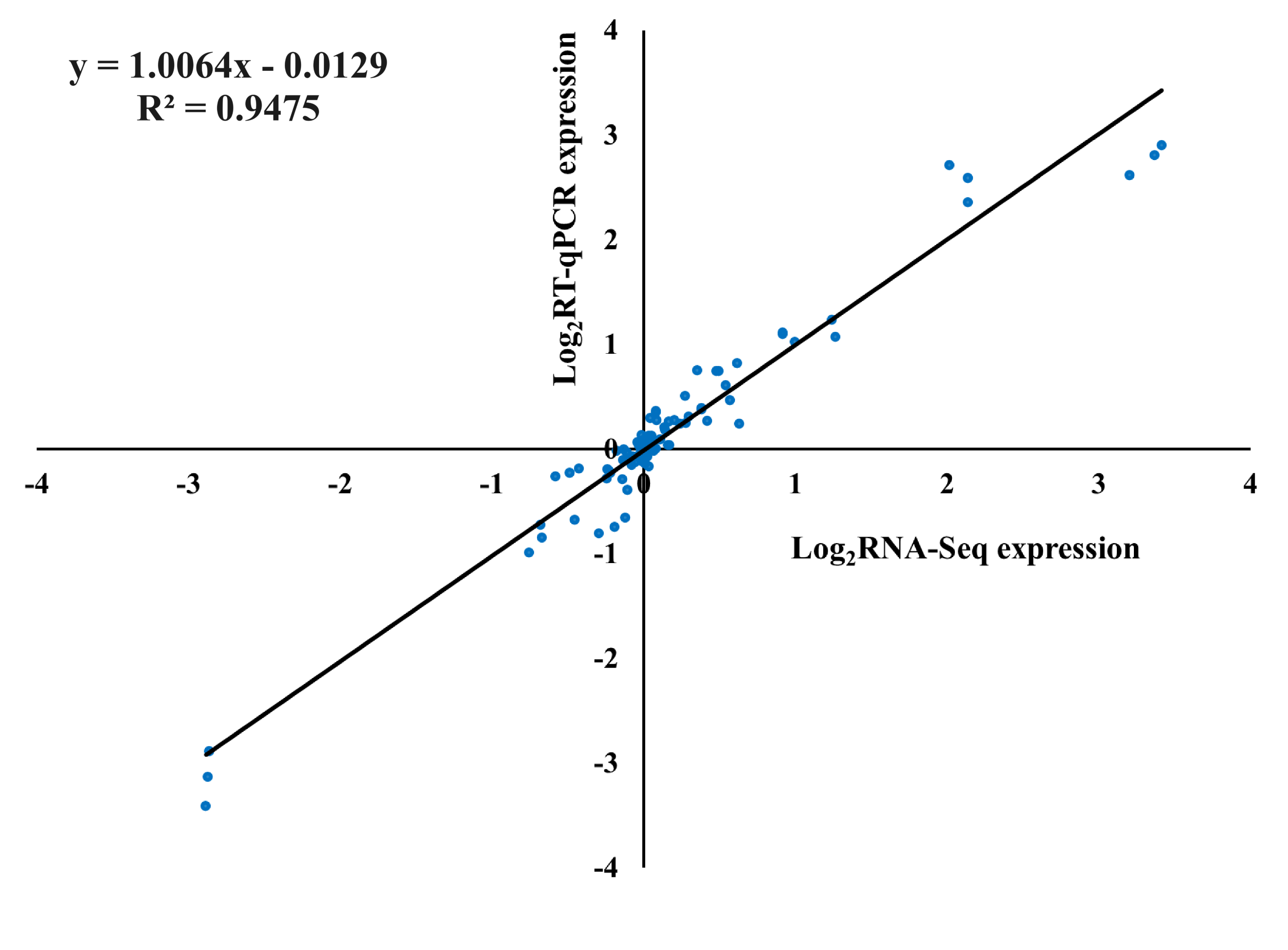 |
| --- |

**Figure S6** Scatter plot showing correlation between gene transcript levels estimated from transcriptome data and those determined by RT-qPCR.
