# Supplementary material for: PtrVINV2 is dispensable for cellulose synthesis but essential for salt tolerance in Populus trichocarpa Torr. and Gray
Source: Plant Biotechnol J. 2025 Feb 24;23(6):1892–908. doi: 10.1111/pbi.70022 (PMC12120930; doi:10.1111/pbi.70022)
Supplement: Supplementary file 7 — Figure S7 Expression patterns of PtrbHLH186, and PtrWND6A genes in PtrVINV2 transgenic plants. OEV and KOV refer to the PtrVINV2‐overexpression and ‐knockout lines, respectively. [file PBI-23-1892-s002.docx]

| 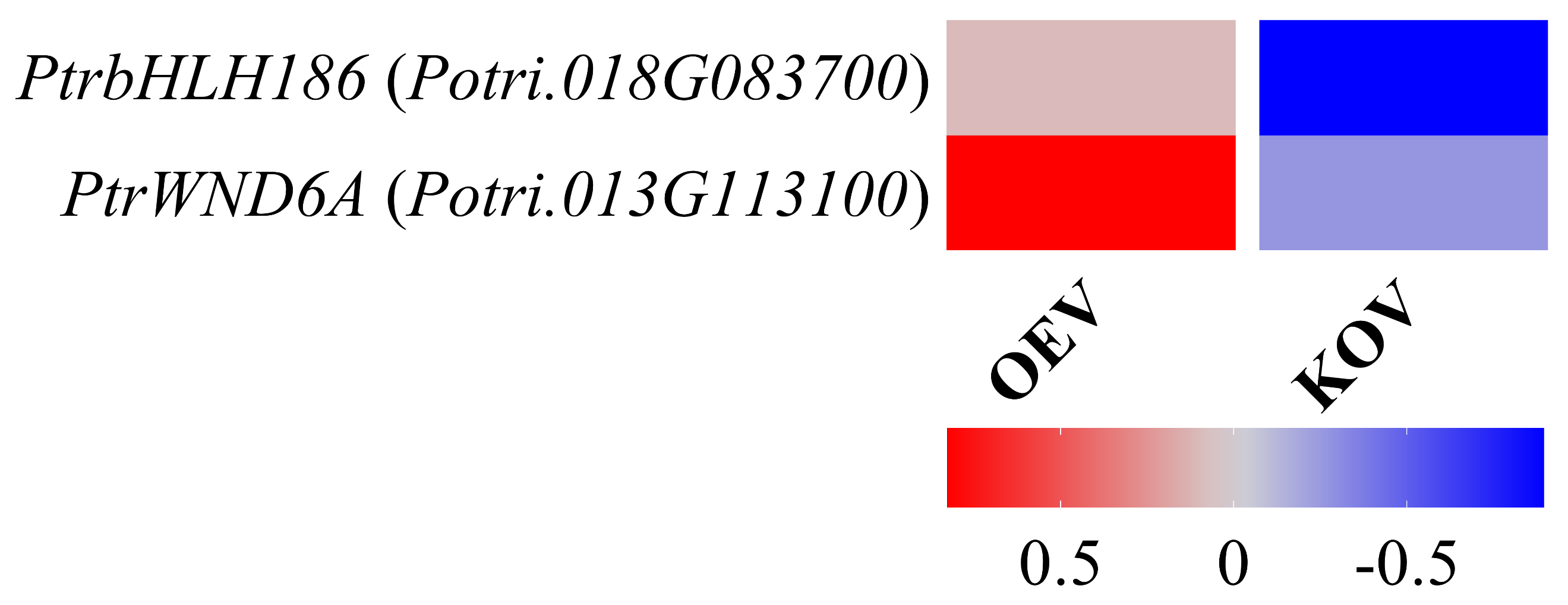 |
| --- |

**Figure S7** Expression patterns of *PtrbHLH186*, and *PtrWND6A* genes in *PtrVINV2* transgenic plants. OEV and KOV refer to the *PtrVINV2*-overexpression and -knockout lines, respectively.
