# Supplementary material for: PtrVINV2 is dispensable for cellulose synthesis but essential for salt tolerance in Populus trichocarpa Torr. and Gray
Source: Plant Biotechnol J. 2025 Feb 24;23(6):1892–908. doi: 10.1111/pbi.70022 (PMC12120930; doi:10.1111/pbi.70022)
Supplement: Supplementary file 8 — Table S1 Connectivity of ‘hub’ transcription factors in the blue, red, turquoise and yellow modules. [file PBI-23-1892-s008.docx]

**Table S1** Connectivity of ‘hub’ transcription factors in the blue, red, turquoise, and yellow modules

| Modules | Gene ID | Type | kTotal | kWithin | kOut | kDiff |
| --- | --- | --- | --- | --- | --- | --- |
| Blue | Potri.019G089000 | bHLH | 1699.61 | 649.15 | 1050.46 | –401.30 |
| Blue | Potri.008G064200 | MYB | 1647.48 | 647.50 | 999.99 | –352.49 |
| Blue | Potri.006G234300 | C3H | 1671.38 | 643.43 | 1027.95 | –384.52 |
| Red | Potri.001G219100 | MYB | 1686.41 | 508.55 | 1177.86 | –669.30 |
| Red | Potri.002G176900 | bHLH | 1660.90 | 491.18 | 1169.71 | –678.53 |
| Red | Potri.001G323500 | CO-like | 1737.48 | 480.43 | 1257.05 | –776.62 |
| Turquoise | Potri.009G106600 | G2-like | 1349.35 | 655.98 | 693.38 | –37.40 |
| Yellow | Potri.009G032900 | GRAS | 1503.53 | 448.61 | 1054.92 | –606.31 |
| Yellow | Potri.002G081000 | NAC | 1276.19 | 448.50 | 827.69 | –379.19 |

Note: kTotal represents the total gene connectivity; kWithin indicates the connectivity within modules; kOut is derived by subtracting kWithin from kTotal; kDiff represents the difference between kWithin and kOut.
