# Supplementary material for: PtrVINV2 is dispensable for cellulose synthesis but essential for salt tolerance in Populus trichocarpa Torr. and Gray
Source: Plant Biotechnol J. 2025 Feb 24;23(6):1892–908. doi: 10.1111/pbi.70022 (PMC12120930; doi:10.1111/pbi.70022)
Supplement: Supplementary file 9 — Table S2 Network connectivity of the PtrVINV2 gene, along with certain genes associated with cellulose, hemicellulose, lignin synthesis and hormone signalling pathways in blue, red, turquoise and yellow modules. [file PBI-23-1892-s006.docx]

**Table S2** Network connectivity of the *PtrVINV2* gene, along with certain genes associated with cellulose, hemicellulose, lignin synthesis, and hormone signaling pathways in blue, red, turquoise, and yellow modules

| Modules | Gene ID | Type | kTotal | kWithin | kOut | kDiff |
| --- | --- | --- | --- | --- | --- | --- |
| Blue | Potri.002G202300 | SUS | 1743.89 | 670.08 | 1073.81 | –403.73 |
| Blue | Potri.012G037200 | SUS | 1207.09 | 423.08 | 784.02 | –360.94 |
| Blue | Potri.004G081300 | SUS | 1367.43 | 501.76 | 865.67 | –363.91 |
| Blue | Potri.005g010800 | NINV | 996.09 | 391.38 | 604.71 | –213.33 |
| Blue | Potri.003g112600 | VINV | 1738.87 | 611.65 | 1127.22 | –515.57 |
| Blue | Potri.003G142300 | CesA | 709.30 | 268.54 | 440.76 | –172.22 |
| Blue | Potri.019G085800 | SUT | 1622.82 | 617.21 | 1005.61 | –388.40 |
| Blue | Potri.003G140900 | UGE | 1386.72 | 511.88 | 874.84 | –362.95 |
| Blue | Potri.008G151100 | STP | 1482.02 | 574.81 | 907.21 | –332.40 |
| Blue | Potri.016G120700 | STP | 936.18 | 347.36 | 588.82 | –241.47 |
| Blue | Potri.006G280700 | ExgA | 420.55 | 143.73 | 276.82 | –133.09 |
| Blue | Potri.006G120000 | CoMT | 1507.64 | 511.52 | 996.12 | –484.60 |
| Blue | Potri.009G139700 | CoMT | 974.04 | 334.04 | 640.01 | –305.97 |
| Blue | Potri.007G122100 | Peroxidase | 1282.58 | 480.36 | 802.22 | –321.86 |
| Blue | Potri.006G129900 | Peroxidase | 1548.09 | 614.09 | 934.01 | –319.92 |
| Blue | Potri.002G018000 | Peroxidase | 1506.01 | 506.54 | 999.47 | –492.93 |
| Blue | Potri.010G078300 | IAA | 556.66 | 206.02 | 350.64 | –144.62 |
| Blue | Potri.006G236200 | IAA | 1510.92 | 578.26 | 932.66 | –354.40 |
| Blue | Potri.003G048100 | IAA | 1382.22 | 531.25 | 850.96 | –319.71 |
| Blue | Potri.002G207800 | TIR1 | 1191.27 | 444.49 | 746.78 | –302.28 |
| Blue | Potri.002G206400 | GH3 | 1568.69 | 541.74 | 1026.96 | –485.22 |
| Blue | Potri.011G129700 | GH3 | 659.05 | 231.88 | 427.17 | –195.287 |
| Blue | Potri.005G096400 | SAUR | 1197.11 | 475.65 | 721.46 | –245.81 |
| Blue | Potri.004G164400 | SAUR | 160.20 | 45.11 | 115.09 | –69.98 |
| Blue | Potri.004G165300 | SAUR | 198.86 | 64.39 | 134.47 | –70.08 |
| Blue | Potri.007G012800 | SAUR | 128.85 | 42.28 | 86.58 | –44.30 |
| Blue | Potri.006G262100 | B-ARR | 1674.45 | 662.38 | 1012.07 | –349.69 |
| Blue | Potri.005G040600 | GID1 | 1589.64 | 599.34 | 990.30 | –390.96 |
| Blue | Potri.013G001300 | PIF3 | 1240.62 | 489.00 | 751.62 | –262.62 |
| Blue | Potri.002G055400 | PIF4 | 1452.66 | 574.54 | 878.13 | –303.60 |
| Blue | Potri.001G092500 | PYL | 1288.73 | 498.38 | 790.35 | –291.96 |
| Blue | Potri.004G138300 | SNRK2 | 258.86 | 91.74 | 167.12 | –75.38 |
| Blue | Potri.006G254100 | EIN3 | 1216.58 | 437.35 | 779.22 | –341.87 |
| Blue | Potri.005G086500 | BIR1 | 1474.06 | 516.75 | 957.31 | –440.57 |
| Blue | Potri.006G043400 | NPR1 | 1536.86 | 558.54 | 978.32 | –419.78 |
| Blue | Potri.002G090700 | TGA | 968.43 | 370.65 | 597.77 | –227.12 |
| Blue | Potri.007G085700 | TGA | 1279.26 | 419.21 | 860.04 | –440.83 |
| Red | Potri.008g101500 | NINV | 1651.75 | 482.22 | 1169.53 | –687.31 |
| Red | Potri.010g236100 | NINV | 318.58 | 53.94 | 264.64 | –210.70 |
| Red | Potri.001G190400 | HXK | 887.05 | 242.40 | 644.65 | –402.25 |
| Red | Potri.006G052600 | CesA | 181.95 | 35.34 | 146.60 | –111.26 |
| Red | Potri.008G148100 | SUT | 364.09 | 97.80 | 266.29 | –168.49 |
| Red | Potri.010G093600 | SUT | 1204.30 | 348.54 | 855.76 | –507.22 |
| Red | Potri.017G029000 | FRK | 1485.39 | 449.70 | 1035.69 | –586.00 |
| Red | Potri.004G117800 | UAM | 1724.81 | 434.69 | 1290.12 | –855.43 |
| Red | Potri.008G192600 | GATL | 1196.30 | 338.07 | 858.23 | –520.16 |
| Red | Potri.010G038300 | GATL | 1697.73 | 502.01 | 1195.72 | –693.71 |
| Red | Potri.015G145400 | PAE | 265.33 | 49.87 | 215.46 | –165.59 |
| Red | Potri.008G060100 | MGP | 801.95 | 194.27 | 607.68 | –413.41 |
| Red | Potri.006G214000 | UXS | 187.95 | 43.51 | 144.44 | –100.93 |
| Red | Potri.004G117800 | UAM | 1724.81 | 434.69 | 1290.12 | –855.43 |
| Red | Potri.011G103700 | RHM | 1384.26 | 406.73 | 977.53 | –570.80 |
| Red | Potri.017G106800 | GAUT | 1142.58 | 326.87 | 815.71 | –488.83 |
| Red | Potri.016G001700 | GAUT | 1355.94 | 349.12 | 1006.82 | –657.71 |
| Red | Potri.004G233500 | STP | 117.62 | 21.77 | 95.84 | –74.07 |
| Red | Potri.012G126100 | TPP | 1700.91 | 511.23 | 1189.68 | –678.45 |
| Red | Potri.006G175500 | TPS | 1659.38 | 451.89 | 1207.49 | –755.60 |
| Red | Potri.004G157100 | ADP-Glc PPase | 227.97 | 67.88 | 160.09 | –92.21 |
| Red | Potri.016G023300 | CAD | 491.55 | 102.73 | 388.82 | –286.09 |
| Red | Potri.005G079200 | PAT | 1052.11 | 273.84 | 778.26 | –504.42 |
| Red | Potri.012G006800 | Peroxidase | 799.18 | 219.50 | 579.68 | –360.18 |
| Red | Potri.008G022700 | Peroxidase | 1530.64 | 322.05 | 1208.60 | –886.55 |
| Red | Potri.016G125000 | Peroxidase | 1354.48 | 407.59 | 946.89 | –539.29 |
| Red | Potri.013G152700 | LAC | 1024.75 | 291.00 | 733.75 | –442.75 |
| Red | Potri.005G237000 | SAUR | 1261.92 | 340.70 | 921.22 | –580.53 |
| Red | Potri.003G113100 | SAUR | 1136.34 | 350.73 | 785.61 | –434.88 |
| Red | Potri.014G164700 | AHK2_3_4 | 1668.64 | 480.13 | 1188.51 | –708.38 |
| Red | Potri.014G136200 | AHP | 305.53 | 44.68 | 260.85 | –216.18 |
| Red | Potri.008G213500 | B-ARR | 1529.68 | 439.81 | 1089.87 | –650.06 |
| Red | Potri.010G001000 | B-ARR | 1266.99 | 356.00 | 910.98 | –554.98 |
| Red | Potri.008G131700 | DELLA | 1462.88 | 420.64 | 1042.24 | –621.61 |
| Red | Potri.009G037300 | PP2C | 598.50 | 151.78 | 446.72 | –294.94 |
| Red | Potri.010G249300 | MKK4_5 | 1431.60 | 355.81 | 1075.79 | –719.98 |
| Red | Potri.014G095500 | JAR | 1513.70 | 450.14 | 1063.56 | –613.42 |
| Red | Potri.018G095100 | CH4 | 1597.71 | 476.98 | 1120.72 | –643.74 |
| Red | Potri.006G071200 | CH4 | 1556.55 | 452.99 | 1103.55 | –650.56 |
| Red | Potri.013G005700 | CH4 | 1484.74 | 415.21 | 1069.53 | –654.32 |
| Red | Potri.003G068900 | JAZ | 1610.61 | 490.75 | 1119.86 | –629.10 |
| Red | Potri.006G139400 | JAZ | 1656.01 | 495.33 | 1160.68 | –665.34 |
| Red | Potri.001G166200 | JAZ | 1670.22 | 496.13 | 1174.09 | –677.96 |
| Red | Potri.001G062500 | JAZ | 1563.78 | 453.60 | 1110.18 | –656.59 |
| Red | Potri.002G176900 | MYC2 | 1660.90 | 491.18 | 1169.71 | –678.53 |
| Red | Potri.001G142200 | MYC2 | 1640.55 | 477.69 | 1162.86 | –685.17 |
| Turquoise | Potri.018G063500 | SUS | 811.87 | 290.31 | 521.56 | –231.26 |
| Turquoise | Potri.008g024100 | NINV | 946.08 | 433.09 | 512.99 | –79.90 |
| Turquoise | Potri.002G197200 | BXL | 970.93 | 474.35 | 496.58 | –22.23 |
| Turquoise | Potri.008G108100 | BXL | 1216.65 | 532.58 | 684.07 | –151.50 |
| Turquoise | Potri.007G129700 | FRK | 361.23 | 137.18 | 224.05 | –86.87 |
| Turquoise | Potri.012G132700 | FRK | 764.40 | 330.18 | 434.23 | –104.05 |
| Turquoise | Potri.019G108900 | GAUT | 240.71 | 77.87 | 162.84 | –84.97 |
| Turquoise | Potri.002G200300 | XS | 1092.95 | 503.92 | 589.04 | –85.12 |
| Turquoise | Potri.005G078500 | ExgA | 1632.53 | 494.27 | 1138.26 | –643.99 |
| Turquoise | Potri.005G166700 | TPP | 831.76 | 364.70 | 467.06 | –102.36 |
| Turquoise | Potri.019G069300 | EG | 1326.45 | 644.80 | 681.65 | –36.86 |
| Turquoise | Potri.005G229700 | ADP-Glc PPase | 769.88 | 255.97 | 513.91 | –257.94 |
| Turquoise | Potri.012G078500 | TPS | 1071.05 | 452.56 | 618.49 | –165.94 |
| Turquoise | Potri.005G052200 | HCT | 82.55 | 26.62 | 55.93 | –29.31 |
| Turquoise | Potri.006G199100 | CAD | 859.61 | 376.00 | 483.60 | –107.60 |
| Turquoise | Potri.001G307200 | CAD | 1086.77 | 382.83 | 703.95 | –321.12 |
| Turquoise | Potri.007G019300 | Peroxidase | 1417.64 | 669.34 | 748.31 | –78.97 |
| Turquoise | Potri.007G096200 | Peroxidase | 1023.41 | 344.76 | 678.65 | –333.88 |
| Turquoise | Potri.003G214700 | Peroxidase | 1485.18 | 593.66 | 891.53 | –297.87 |
| Turquoise | Potri.004G052100 | Peroxidase | 825.65 | 391.68 | 433.97 | –42.29 |
| Turquoise | Potri.003G214800 | Peroxidase | 1517.65 | 474.16 | 1043.50 | –569.34 |
| Turquoise | Potri.003G214900 | Peroxidase | 943.03 | 397.51 | 545.52 | –148.02 |
| Turquoise | Potri.016G112100 | LAC | 1110.04 | 513.09 | 596.96 | –83.87 |
| Turquoise | Potri.006G098300 | AUX1 | 1414.53 | 652.56 | 761.96 | –109.40 |
| Turquoise | Potri.008G161200 | IAA | 689.29 | 314.95 | 374.34 | –59.39 |
| Turquoise | Potri.014G100100 | ARF | 1128.82 | 489.79 | 639.03 | –149.23 |
| Turquoise | Potri.004G050150 | ARF | 1593.32 | 559.00 | 1034.33 | –475.33 |
| Turquoise | Potri.009G125900 | SAUR | 940.94 | 480.86 | 460.09 | 20.77 |
| Turquoise | Potri.018G063400 | SAUR | 726.51 | 339.15 | 387.36 | –48.21 |
| Turquoise | Potri.010G102900 | AHK2_3_4 | 1500.28 | 470.76 | 1029.53 | –558.77 |
| Turquoise | Potri.008G137900 | AHK2_3_4 | 1155.17 | 521.95 | 633.22 | –111.27 |
| Turquoise | Potri.005G040400 | AHP | 607.24 | 311.99 | 295.26 | 16.73 |
| Turquoise | Potri.018G111300 | B-ARR | 432.19 | 156.04 | 276.15 | –120.11 |
| Turquoise | Potri.016G038000 | A-ARR | 1383.75 | 668.28 | 715.47 | –47.20 |
| Turquoise | Potri.010G037800 | A-ARR | 1136.68 | 334.80 | 801.88 | –467.08 |
| Turquoise | Potri.001G027000 | A-ARR | 468.47 | 197.29 | 271.18 | –73.90 |
| Turquoise | Potri.008G193000 | A-ARR | 1414.86 | 685.55 | 729.31 | –43.76 |
| Turquoise | Potri.002G082200 | A-ARR | 1299.68 | 642.17 | 657.51 | –15.34 |
| Turquoise | Potri.014G022100 | GID2 | 1321.72 | 456.65 | 865.07 | –408.42 |
| Turquoise | Potri.014G097100 | PYL | 476.12 | 208.91 | 267.21 | –58.30 |
| Turquoise | Potri.008G073400 | PYL | 371.57 | 141.56 | 230.01 | –88.46 |
| Turquoise | Potri.010G183900 | PYL | 331.05 | 82.04 | 249.01 | –166.96 |
| Turquoise | Potri.001G245200 | PP2C | 197.54 | 70.62 | 126.92 | –56.29 |
| Turquoise | Potri.008G010800 | ABF | 612.25 | 266.24 | 346.01 | –79.77 |
| Turquoise | Potri.009G142800 | BSK | 106.07 | 43.12 | 62.95 | –19.83 |
| Turquoise | Potri.014G179300 | BSK | 74.30 | 19.31 | 54.99 | –35.69 |
| Turquoise | Potri.007G048300 | CYCD3 | 1023.26 | 519.88 | 503.38 | 16.51 |
| Turquoise | Potri.010G108200 | JAZ | 1431.25 | 590.20 | 841.05 | –250.85 |
| Yellow | Potri.011G069600 | CesA | 1040.33 | 308.06 | 732.27 | –424.21 |
| Yellow | Potri.008G094300 | UGD | 1030.62 | 265.75 | 764.86 | –499.11 |
| Yellow | Potri.002G132900 | GATL | 1483.94 | 440.76 | 1043.19 | –602.43 |
| Yellow | Potri.007G031700 | GATL | 1490.55 | 423.44 | 1067.10 | –643.66 |
| Yellow | Potri.001G068100 | IRX | 1238.72 | 424.27 | 814.45 | –390.18 |
| Yellow | Potri.012G109200 | IRX | 1296.05 | 341.50 | 954.56 | –613.06 |
| Yellow | Potri.012G109600 | IRX | 1116.78 | 293.37 | 823.40 | –530.03 |
| Yellow | Potri.011G132600 | IRX | 1374.32 | 406.06 | 968.26 | –562.20 |
| Yellow | Potri.006G131000 | IRX | 987.68 | 310.15 | 677.53 | –367.38 |
| Yellow | Potri.001G237200 | UXS | 232.04 | 70.62 | 161.42 | –90.79 |
| Yellow | Potri.006G022000 | UAE | 904.49 | 254.59 | 649.90 | –395.30 |
| Yellow | Potri.006G001100 | GAUT | 1304.97 | 392.07 | 912.91 | –520.84 |
| Yellow | Potri.010G089800 | STP | 961.56 | 335.54 | 626.02 | –290.48 |
| Yellow | Potri.008G038200 | PAL | 616.53 | 144.04 | 472.49 | –328.45 |
| Yellow | Potri.016G091100 | PAL | 854.25 | 221.09 | 633.16 | –412.07 |
| Yellow | Potri.010G224100 | PAL | 1387.31 | 419.22 | 968.09 | –548.87 |
| Yellow | Potri.013G157900 | C4H | 1060.10 | 266.86 | 793.24 | –526.38 |
| Yellow | Potri.019G130700 | C4H | 1070.08 | 294.17 | 775.91 | –481.74 |
| Yellow | Potri.016G031100 | C3H | 790.21 | 209.84 | 580.38 | –370.54 |
| Yellow | Potri.006G033300 | C3H | 1170.83 | 367.31 | 803.51 | –436.20 |
| Yellow | Potri.013G122000 | CoMT | 1082.48 | 223.59 | 858.89 | –635.30 |
| Yellow | Potri.001G451100 | CoMT | 874.42 | 258.22 | 616.20 | –357.98 |
| Yellow | Potri.009G063300 | CAD | 909.48 | 345.04 | 564.44 | –219.40 |
| Yellow | Potri.001G268600 | CAD | 1133.10 | 364.41 | 768.68 | –404.27 |
| Yellow | Potri.006G024300 | CAD | 620.52 | 175.46 | 445.07 | –269.61 |
| Yellow | Potri.009G148800 | ADT | 830.09 | 217.12 | 612.98 | –395.86 |
| Yellow | Potri.001G011500 | Peroxidase | 606.18 | 186.63 | 419.55 | –232.92 |
| Yellow | Potri.017G064100 | Peroxidase | 432.77 | 143.36 | 289.42 | –146.06 |
| Yellow | Potri.001G182400 | Peroxidase | 688.44 | 107.13 | 581.31 | –474.18 |
| Yellow | Potri.006G087100 | LAC | 1183.46 | 405.51 | 777.95 | –372.44 |
| Yellow | Potri.001G054600 | LAC | 931.36 | 312.90 | 618.46 | –305.56 |
| Yellow | Potri.001G401300 | LAC | 754.51 | 237.79 | 516.72 | –278.93 |
| Yellow | Potri.009G156600 | LAC | 970.91 | 362.13 | 608.78 | –246.65 |
| Yellow | Potri.011G120200 | LAC | 490.72 | 166.65 | 324.07 | –157.42 |
| Yellow | Potri.019G088900 | LAC | 723.51 | 217.91 | 505.59 | –287.68 |
| Yellow | Potri.009G156800 | LAC | 1119.53 | 407.48 | 712.06 | –304.58 |
| Yellow | Potri.001G184300 | LAC | 1269.52 | 448.78 | 820.75 | –371.97 |
| Yellow | Potri.008G064000 | LAC | 1102.31 | 391.38 | 710.94 | –319.56 |
| Yellow | Potri.010G183500 | LAC | 1005.26 | 297.64 | 707.63 | –409.99 |
| Yellow | Potri.016G112000 | LAC | 1309.17 | 443.99 | 865.17 | –421.18 |
| Yellow | Potri.001G298300 | GH3 | 1384.92 | 353.80 | 1031.12 | –677.33 |
| Yellow | Potri.014G136800 | GH3 | 873.63 | 289.85 | 583.78 | –293.93 |
| Yellow | Potri.006G126500 | SAUR | 819.93 | 244.26 | 575.67 | –331.42 |
| Yellow | Potri.006G211000 | SAUR | 919.88 | 224.87 | 695.01 | –470.14 |
| Yellow | Potri.002G145300 | SAUR | 200.19 | 63.75 | 136.44 | –72.69 |
| Yellow | Potri.003G171000 | AHK2_3_4 | 921.02 | 241.49 | 679.53 | –438.04 |
| Yellow | Potri.008G135500 | B-ARR | 888.65 | 151.73 | 736.91 | –585.18 |
| Yellow | Potri.003G084100 | SNRK2 | 1226.14 | 423.00 | 803.14 | –380.14 |
| Yellow | Potri.005G223200 | ERF1 | 616.63 | 185.36 | 431.27 | –245.91 |
| Yellow | Potri.002G011800 | BSK | 1518.98 | 422.58 | 1096.40 | –673.82 |
| Yellow | Potri.005G249300 | BSK | 444.91 | 105.06 | 339.85 | –234.78 |
