# Supplementary material for: PtrVINV2 is dispensable for cellulose synthesis but essential for salt tolerance in Populus trichocarpa Torr. and Gray
Source: Plant Biotechnol J. 2025 Feb 24;23(6):1892–908. doi: 10.1111/pbi.70022 (PMC12120930; doi:10.1111/pbi.70022)
Supplement: Supplementary file 10 — Table S3 Sequences of all primers used in this study. [file PBI-23-1892-s003.docx]

**Table S3** Sequences of all primers used in this study

| **Primers required for vector construction** | | | |  |
| --- | --- | --- | --- | --- |
| **Primers name** | **Primers sequence (5′-3′)** | | **Remarks** |  |
| PtrVINV2-CDS-F | CTCCCGTTTACGCCTACCAT | | Primers used for cloning the full-length sequence of the *PtrCWINV3* | |
| PtrVINV2-CDS-R | AGGCATTGCTACTGTTGTTCA | |  |  |
| PtrVINV2-pBS-F  PtrVINV2-pBS-R | GCTCTAGAATGGCGGACCCAAGTC  GGACTAGTCTGTTGTTCATTAGAATATGGGC | | Primers used for subcellular Localization vector construction | |
| DX15-F  DX15-R | TCCCCCTTTTGGTTCAATGC  TTGTGGCCTCATCTTGAAGTA | | Primers used for cloning the DX15 promoter | |
| PtrVINV2-Xba I-F  PtrVINV2-Kpn I-R | GCTCTAGAGCATGGCGGACCCAAGTCCATT  GCTCTAGACTACTTGTCATCGTCGTCCTTGTAGTCGTCCTGTTGTTCATTAGAAT | | Primers used for overexpression vector construction | |
| PtrVINV2-DT1-BsF  PtrVINV2-DT1-F0  PtrVINV2-DT2-R0  PtrVINV2-DT2-BsR | ATATATGGTCTCGATTGGTGAATTTGAAGGGAGCTCGTT  TGGTGAATTTGAAGGGAGCTCGTTTTAGAGCTAGAAATAGC  AACGTGTATAGCATGACGATCTCAATCTCTTAGTCGACTCTAC  ATTATTGGTCTCGAAACGTGTATAGCATGACGATCTCAA | | Primers used for CRISPR/Cas9 vector construction | |
| PtrVINV2-DT3-BsF  PtrVINV2-DT3-F0  PtrVINV2-DT4-R0  PtrVINV2-DT4-BsR | ATATATGGTCTCGATTGGCCGTCTCAGCCGGATGTC GTT  TGGCCGTCTCAGCCGGATGTC GTTTTAGAGCTAGAAATAGC  AACTGGAACAATAGCATGTTATCAATCTCTTAGTCGACTCTAC  ATTATTGGTCTCGAAACTGGAACAATAGCATGTTATCAA | |  |  |
| **Primers Required for Assessing Gene Editing in Knockout Plants** | | | |  |
| **Primers name** | **Primers sequence (5′-3′)** | | **Remarks** |  |
| PtrVINV2-DT1/2-Cas9-F | ggagtgagtacggtgtgcCCGAGACTTTGTTGCCAGCAG | | Primers used for sequencing on the Hi-TOM platform |  |
| PtrVINV2-DT1/2-Cas9-R | gagttggatgctggatggCGTTCATCCAATTCTCTTCAGGT | |  |  |
| PtrVINV2-DT3-Cas9-F | ggagtgagtacggtgtgcTCCTCCCTGATGGTAAGATCGTC | |  |  |
| PtrVINV2-DT3-Cas9-R | gagttggatgctggatggAGTTTTCCAGGCTGTTGTGGG | |  |  |
| PtrVINV2-DT4-Cas9-F | ggagtgagtacggtgtgcATAGTCCTGCCCCAGAAGGG | |  |  |
| PtrVINV2-DT4-Cas9-R | gagttggatgctggatggTGACACACCGGCAGATACCC | |  |  |
| **Primers for qRT-PCR** | | | |  |
| **Gene name** | **Primers sequence (5′-3′)** | | **Remarks** |  |
| Potri.018G063500 | F: AAAACATTGGCAGGCGGTGC  R: GCACGAAGTTGCCGTTGCAG | Primers needed for validating transcriptomic data | |  |
| Potri.006G136700 | F: AAGTGCAAGGCTGATCCCAGT  R: AGATAGCGACGGCTCTCACGA |  |  |  |
| Potri.008g101500 | F: TTGATTCTGGGCTGTGGTGG  R: CCGAGTCTGATTCCTGTCTGG |  |  |  |
| Potri.006G210600 | F: GGCGGGGCTCAGATATGATT  R: CAGACTCGTTAGCCCAACCC |  |  |  |
| Potri.003g112600  (*PtrVINV2*) | F: GCCACAATCCTCCCTGATGG  R: GGATCATCGTGGTCTGCTGG |  |  |  |
| Potri.009G050000 | F: GCTGGCTGCAATGCATGAGG  R: TCCACTTCCATCCCGTCCGA |  |  |  |
| Potri.004G059600 | F: CGGAACAGCAGATGGAAGATA  R: CCAGAATGACCAGTCGCATAA |  |  |  |
| Potri.013G115200 | F: TCTAACACTTCGGGTGCTGG  R: TACTGGTGGGAGCCCTCTTG |  |  |  |
| Potri.004G118600 | F: TCCACAGGTCCAGAAAGAACA  R: TTGCTCAACAGCAGAGCTGG |  |  |  |
| Potri.010G089800 | F: CCTGTCGGAGGGTTAGCG  R: CAAGCCAGCCAAGTAAAGCG |  |  |  |
